# Ensemble learning from ensemble docking: revisiting the optimum ensemble size problem

**DOI:** 10.1038/s41598-021-04448-5

**Published:** 2022-01-10

**Authors:** Sara Mohammadi, Zahra Narimani, Mitra Ashouri, Rohoullah Firouzi, Mohammad Hossein Karimi‐Jafari

**Affiliations:** 1 Department of Bioinformatics, Institute of Biochemistry and Biophysics, University of Tehran, Tehran, Iran; 2 Department of Computer Science and Information Technology, Institute for Advanced Studies in Basic Sciences (IASBS), 45137-66731 Zanjan, Iran; 3 Department of Physical Chemistry, Chemistry and Chemical Engineering Research Center of Iran, Tehran, Iran

**Keywords:** Computational platforms and environments, Machine learning, Virtual drug screening

## Abstract

Despite considerable advances obtained by applying machine learning approaches in protein–ligand affinity predictions, the incorporation of receptor flexibility has remained an important bottleneck. While ensemble docking has been used widely as a solution to this problem, the optimum choice of receptor conformations is still an open question considering the issues related to the computational cost and false positive pose predictions. Here, a combination of ensemble learning and ensemble docking is suggested to rank different conformations of the target protein in light of their importance for the final accuracy of the model. Available X-ray structures of cyclin-dependent kinase 2 (CDK2) in complex with different ligands are used as an initial receptor ensemble, and its redundancy is removed through a graph-based redundancy removal, which is shown to be more efficient and less subjective than clustering-based representative selection methods. A set of ligands with available experimental affinity are docked to this nonredundant receptor ensemble, and the energetic features of the best scored poses are used in an ensemble learning procedure based on the random forest method. The importance of receptors is obtained through feature selection measures, and it is shown that a few of the most important conformations are sufficient to reach 1 kcal/mol accuracy in affinity prediction with considerable improvement of the early enrichment power of the models compared to the different ensemble docking without learning strategies. A clear strategy has been provided in which machine learning selects the most important experimental conformers of the receptor among a large set of protein–ligand complexes while simultaneously maintaining the final accuracy of affinity predictions at the highest level possible for available data. Our results could be informative for future attempts to design receptor-specific docking-rescoring strategies.

Intermolecular interactions are the basis of almost all biological processes, and different experimental methods and computational techniques have been developed to analyze them. Specifically, in the case of healing treatments, structure-based drug design attempts to design candidate drugs according to their interaction with the three-dimensional structure of the target proteins^1–3^. In this direction, docking technologies have been improved considerably with two main goals: accurate prediction of the geometry of binding (pose prediction) and reliable scoring of different binding geometries (affinity prediction)^4–6^. Many docking software programs have also been developed in which each of them adopts one or more different binding paradigms from the earliest lock-and-key models to more sophisticated search and scoring conformational selection strategies^7,8^. These tools have been the subject of continuous assessments over many years regarding their ability for pose and affinity prediction. Two widely used free and open-source docking packages are Autodock4 (AD4)^9^ and Autodock Vina (Vina)^10^, with thousands of citations and many cases of successful discovery stories since their initial release dates^11^. A recent comparative investigation benchmarked these two packages on a diverse set of protein − ligand complexes^12^. The results show that in general, Vina reproduces more accurate binding poses, while AD4 gives binding affinities that correlate better with experimental values^12^. However, the results are receptor dependent, and for a specific target, it seems that a specific benchmarking is desirable to decide on the best docking tool.

The flexibility of the receptor and the way of incorporating it in the docking procedure has remained the most challenging problem in protein-small molecule studies. It is now a well-known fact that proteins should be considered as an ensemble of conformations not only for intrinsically disordered cases but also for describing native states of ordered globular ones^13,14^. The dynamics of proteins over their conformational ensembles enable them to harness thermodynamic fluctuations for specific recognition of their targets, including small molecules^15^. This diversity adds an additional search dimension in docking procedures, and as a result, different approaches have been designed to incorporate receptor flexibility through ensemble docking^16,17^. A receptor ensemble can be constructed computationally via molecular dynamics simulations or other phase space sampling methods^18–21^. The alternative is using available experimental structures of the receptor reported in complex with different small molecules at the binding site. Currently, for some important proteins, there are hundreds of reported X-ray structures in the protein data bank that provide an informative image of the conformational diversity of bound states of that receptor. However, the main challenge is how to select a representative set of structures for an efficient docking simulation considering the fact that the computational cost of ensemble docking increases with the number of receptor conformations. Another challenge in ensemble docking is the problem of false positive predictions that generally show an increased rate with the enlargement of the receptor ensemble^22,23^.

Even with a perfect set of receptor conformations and corresponding binding geometries, their correct scoring would be the bottleneck of a pipeline for reliable pose or affinity prediction. Many scoring functions have been developed in recent years, and some of them have been revisited with the considerable increase in available experimental geometry and affinity data^24–26^. Three conventional schemes, including force-field, knowledge-based and empirical scoring functions, have shown severe limitations in their potential for improvement with the rapid growth of training data^6,24,27,28^. Although these schemes differ from each other in how they consider well-defined physically meaningful contributions of protein–ligand interactions, all of them are restricted to a parametric form of a linear combination of these contributions. On the other hand, modern machine learning scoring functions go beyond these additive and linear assumptions, and with the increase in available experimental data, it seems that they can provide more promising solutions not only for the case of protein–ligand interactions but also for other cases of molecular recognition in biological systems^29–31^. Different machine learning methods have been applied to affinity prediction problems, including support vector regression, artificial neural networks, random forest (RF), and boosted regression trees (BRT)^32–35^. Comparison of the linear and nonlinear regression over training datasets of different sizes showed that the efficiency of linear methods remained constant, while the efficiency of the RF method magnificently increased^29,36^. In another assessment that compared 16 conventional and six machine learning scoring functions, it was shown that ensemble prediction methods such as RF and BRT outperform other scoring schemes. The authors also reported steady gains in the performance of these two methods as the training set size and type and number of features were increased^37^.

Notably, both well-performing methods, RF and BRT, belong to the ensemble learning paradigm in supervised learning. As a machine learning counterpart for the so-called wisdom of the crowd, ensemble learning methods combine the results of multiple base learners (decision trees in the case of RF and BRT) to compensate for the errors of each learner via a weighting and aggregating procedure^36–38^. The improved predictive performance of ensemble learning methods is a result of avoiding overfitting of a single learner in the case of a small amount of data. Moreover, they avoid trapping in a local optimum and provide an extended representation of the problem beyond the space of any single model. Another advantage related to the goal of the current study is tackling the curse of dimensionality. For a fixed size of data and a large number of features, single learners become less generalizable, while some of the ensemble methods can lessen the problem via attribute bagging^38^. From a point of view, different ensemble learning methods can be classified as bagging or boosting. In 1994, the bagging idea, presented by Breiman, was retrieved from bootstrap aggregating meaning^39^. In bagging algorithms, a dataset is sampled with replacement to create several subsets of the original data. The learning process is applied to each subset, resulting in a collection of models. The prediction (or regression) process uses the maximum vote (or output mean) of the models, resulting in a lower variance of the overall model. RF is a popular bagging-based method. The boosting method refers to an effective method of combining a set of weak learners, with an emphasis on previously misclassified samples in each learner, to achieve one strong learner^40^.

In this study, we combined ensemble learning methods with the ensemble docking procedure to address the problem of optimum selection from a set of X-ray structures and to keep the computational cost and false positive prediction rates as low as possible while achieving a receptor-specific affinity predictor as accurate as possible. In this procedure, cyclin-dependent kinase 2 (CDK2) was selected as a test case. This enzyme belongs the serine/threonine kinases family and has the main role in controlling the cell cycle and meiosis. CDK2 plays a role in balancing cell proliferation, cell death and DNA restoration in human embryonic stem cells^41^. The activity of this kinase is regulated through the binding of cyclin subunits and phosphorylation at specific sites^42^. Regarding its role in cancer cell proliferation^43^, inhibition of CDK2 in its free or cyclin-bound states has been the subject of many experimental and computational studies^44^. Accordingly, the availability of numerous ligand-bound X-ray structures and independent measured binding affinity values for many drug-like molecules makes CDK2 an ideal test case for the current study.

## Results and discussions

### Diversity of CDK2 structures

**Figure 1:**
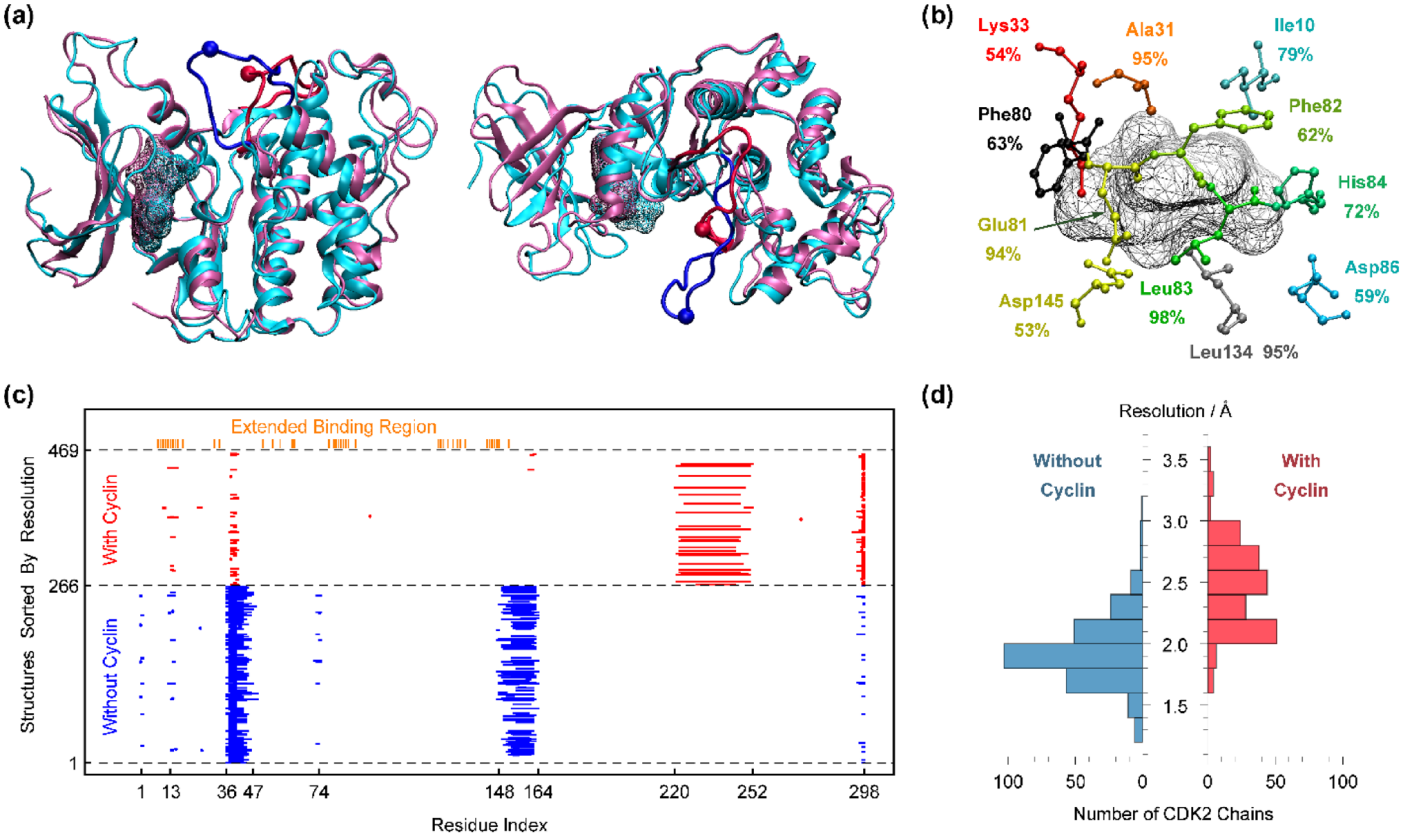
(**a**) Side and top views of two representative CDK2 structures (1FQ1B in cyan and 1JSTA in pink). ATP molecules in binding sites are in wireframe. The activation loop is colored in blue (1FQ1B) or red (1JSTA) with a bead in position of Thr160 (1FQ1B) or its phosphorylated form Tpo160 (1JSTA). (**b**) Extended binding region around ATP molecule (1JSTA). Only residues with more than 50% ligand contact among 315 structures were depicted. (**c**) Pattern of missing residues in initial receptor ensemble of CDK2 chains and position of residues in extended binding region along sequence. (**d**) Resolution of X-ray CDK2 structures complexed with or without cyclin.

CDK2 has a bilobal structure enriched with beta-sheets in the N-terminal domain (small lob) and alpha helices in the C-terminal domain (large lob)^42^ (Fig. 1a). The ATP binding site (Fig. 1b) is the main target of most inhibition studies^44^. The presence or absence of cyclin induces a conformational change in CDK2, as highlighted for two representative structures in Fig. 1a. Most affected is an intrinsically disordered segment denoted as the activation loop, which is relocated in the presence of cyclin. The functionally important residue Thr160 is also located in this segment, and its phosphorylation can further stabilize the cyclin-free configuration^43^. Due to dynamic disorder, this loop and some other parts of the sequence have been reported as missing residues in many structures. As shown in Fig. 1c, the pattern of missing residues becomes completely different in the presence of cyclin. Two segments corresponding to residues 35–47 and 147–164 have some missing residues in almost all structures in the absence of cyclin, while a long segment around 220–252 has some missing residues in many CDK2 structures complexed with cyclin. Since in each part of Fig. 1c the structures are sorted by reported resolution, it can be said that these patterns of disordered segments do not depend on the quality of X-ray structures although cyclin-free CDK2 chains have a better resolution in comparison with cyclin-bound cases (Fig. 1d).

Among 315 chains in the curated receptor ensemble (listed in Table S1), 26 chains contain an ATP/ADP molecule, and the remaining chains contain a diverse set of coccrystallized small molecules in the ATP binding site or its vicinity (see Table S1). To be generalizable for subsequent applications, the extended binding pocket (Fig. 1c) was defined by considering all 41 residues that are in contact with any ligand in any receptor (see Table S2). Among them, 11 residues have contact with more than 50% of ligands. The most frequent binding residues are Leu83, Ala31, Leu134 and Glu81, which are in contact with ligands in 98%, 95%, 95% and 94% of complexes, respectively. However, in our strategy, every residue matters, even those 6 residues that show only one contact with a single ligand in all curated structures (see Table S2).

### Clustering vs. graph-based redundancy removal

**Figure 2:**
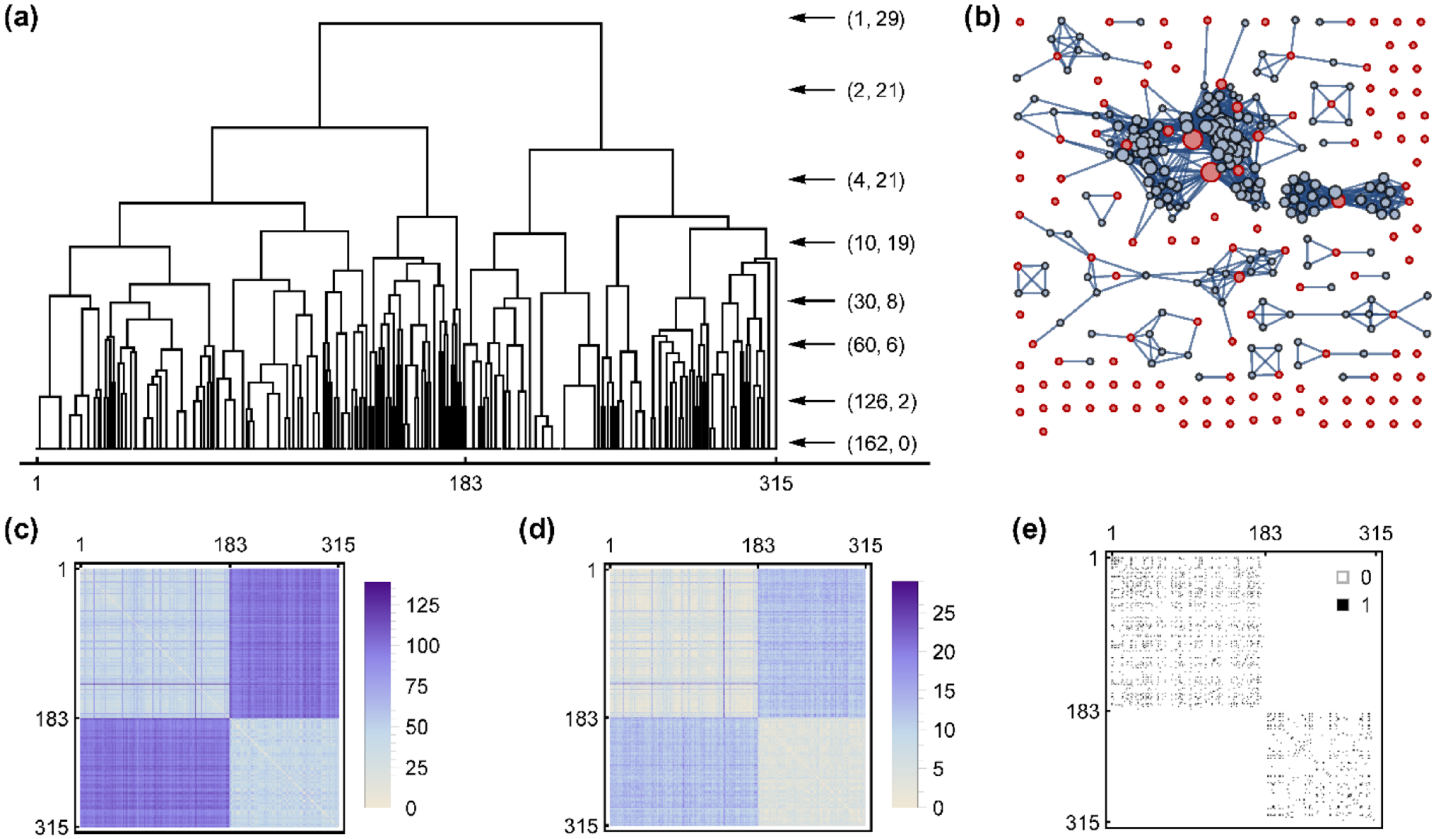
(**a**) Hierarchical clustering of curated receptor ensemble based on dissimilarity matrix ${\varvec{N}}$. For better presentation a logarithmic scale was used vertically. The arrows point to some possible levels of dendrogram truncation for which the first number in parenthesis is the number of clusters and the second one is the maximum intra-cluster dissimilarity. (**b**) Redundancy graph of curated receptor ensemble. Nodes are receptors scaled by their centrality and edges are redundancies (${N}_{ij}=0$ or ${A}_{ij}=1$). Red nodes are 126 members of non-redundant receptor ensemble selected via an iterative centrality-based procedure. (**c**) Dissimilarity matrix ${\varvec{T}}$. (**d**) Dissimilarity matrix ${\varvec{N}}$. (**e**) Adjacency matrix ${\varvec{A}}$. In all plots, the first 183 receptors are without cyclin.

The size of the curated receptor ensemble is much larger than that applicable in an ensemble docking strategy. In addition to the computational cost, it has been shown that a larger receptor ensemble increases the rate of false positive predictions in ensemble docking^45,46^. The routine solution is choosing a reasonably small set of structures via a representative selection strategy, which is almost always clustering based on the RMSD measure over the relevant part of the structure, i.e., the ligand binding region. Such a “cluster-based” and “average-preferred” way of thinking loses less frequent patterns of binding reflected in the conformational diversity of the binding region. Moreover, an all-at-once calculation of RMSD, for all important residues, has the known disadvantage of diminishing rare but important differences of some residues in the “mean” character of RMSD^47^. To avoid this problem, ${T}_{ij}$ (Eq. 2) and ${N}_{ij}$ (Eq. 3) dissimilarity matrices were introduced and used in a routine clustering approach (see Fig. 2). Splitting all receptors into two clusters (the second uppermost arrow in Fig. 2a) separates the free CDK2 chains from cyclin-bound chains. Thus, even in the extended binding region and irrespective of the type of ligand, the main factor of structural diversity is the availability of cyclin. This nontrivial fact is also reflected in the structure of dissimilarity matrices (Figs. 2c,d) and the distribution of their elements (Fig. S1).

Indeed, choosing two representative receptors from two clusters of free and cyclin-bound CDK2 is not reasonable since the maximum intracluster dissimilarity is 21. In other words, in one of the clusters, there are two receptors that differ from each other in 21 residues of the extended binding region. Other arrows in Fig. 2a show how the heterogeneity of clusters is reduced by increasing the number of clusters. This heterogeneity affects the meaning of the “representative” structure and shows the extent of ignored diversity of binding patterns below a truncation level. Even in the case of 60 clusters, at least one cluster has two structures that differ from each other in 6 residues of the extended binding region. Moreover, prior selection of a specific truncation level is to some extent an arbitrary subjective choice without other stifications. In this regard, we decided to let machine learning not only rescores the results of ensemble docking but also decides which members of the curated receptor ensemble are more important to be included in the docking ensemble. The suggested approach has less arbitrariness and puts the selection of receptor structures in an objective perspective based on the final performance of ensemble learning.

However, it is wise to remove redundant structures via a redundancy criterion before performing docking simulations. Based on the well-known 2 Å cutoff for RMSD, two receptors were considered the same if none of their residues in the extended binding region had RMSD values larger than 2 Å (i.e., ${N}_{ij}=0$). The lowermost arrow in Fig. 2a shows that at least 162 clusters should be considered to reach a level for which all members but one within any cluster are redundant (all intracluster ${N}_{ij}=0$). This number could depend on the adapted clustering algorithm or its specific features (for example Ward linkage used here). As an alternative, a robust graph-based redundancy removal is suggested here that does not depend on any user-defined parameter except the 2 Å cutoff in the definition of ${N}_{ij}$. The corresponding redundancy graph built from the ${A}_{ij}$ matrix (Eq. 3 and Fig. 2e) is depicted in Fig. 2b with 126 selected nonredundant receptors highlighted. The iterative procedure of selection based on graph centrality is justified by design since it considers the adjacencies among receptors. The number of nonredundant receptors, 126, is the possible minimum that is smaller than the number obtained from clustering, 162, which needs some postprocessing to remove redundancies between cluster representatives.

### Orthosteric, allosteric and optimum docking regions

**Figure 3:**
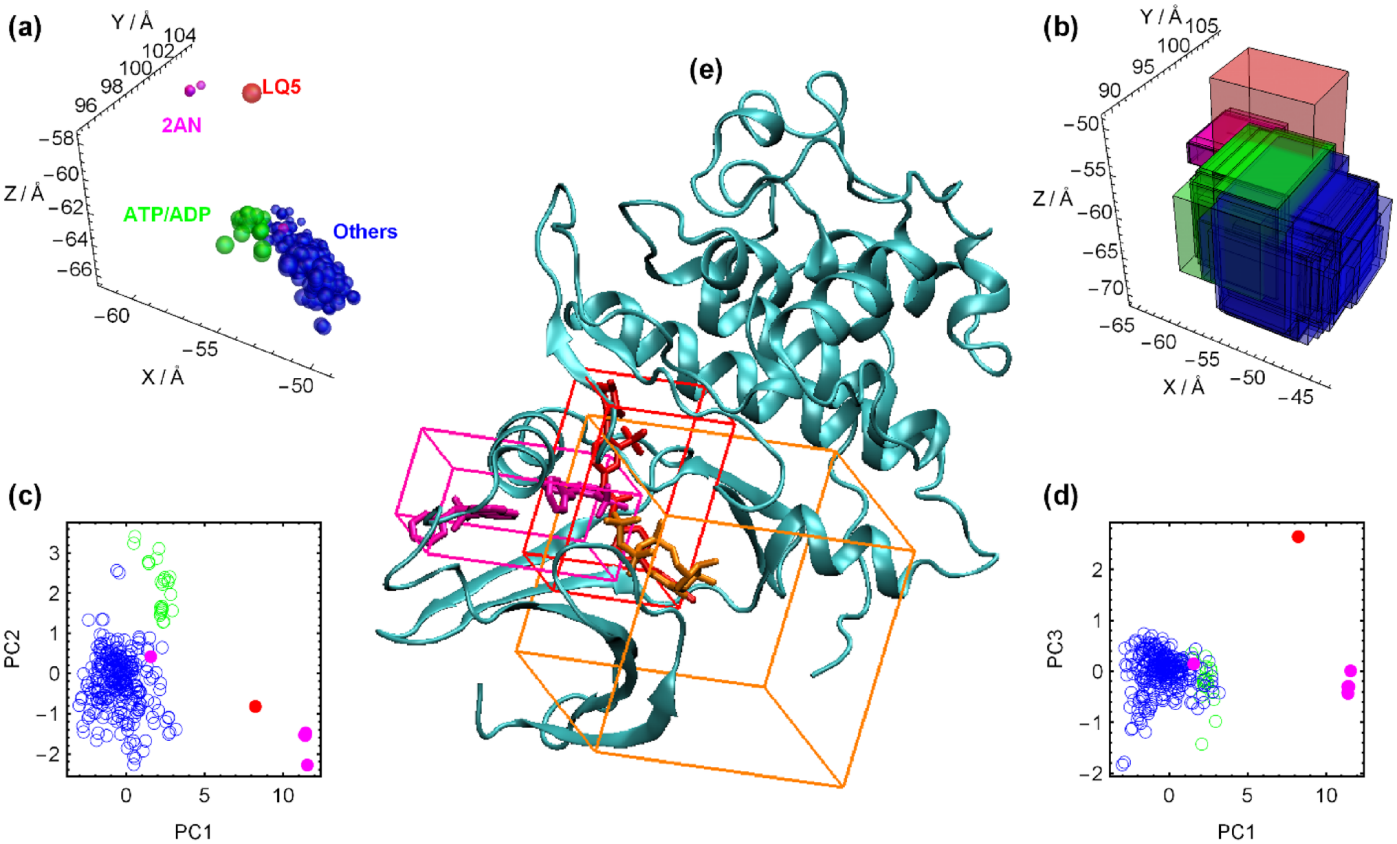
(**a**) Center of geometry of ligands in CDK2 structures. Size of spheres are rescaled relative to the ligand size. (**b**) Spatial extension of ligand enclosing boxes. (**c**,**d**) Three principal components of ligand centers. (**e**) Position of common docking box (orange) used for all receptors on a representative structure (1JSTA) including ATP molecule compared to bounding boxes of LQ5 (red) and 2AN ligands in the allosteric site (magenta).

Since the aim of the current study is ML- and structure-based rescoring of docking poses in conjunction with ML-derived importance of receptor structures, the correct definition of docking box plays a vital role that affects the generalizability and robustness of the whole pipeline. As shown in Fig. 3, most cocrystallized ligands (except 2AN and LQ5) are located near the position of ATP/ADP molecules, although their positions differ slightly, as reflected by enclosing boxes, geometric centers and their principal components. Some ligands do not penetrate completely in the ATP binding pocket. This space was considered the orthosteric site and must be covered in docking. On the other hand, behind the orthosteric pocket, an allosteric site with separate entry has been suggested based on the binding behavior of 8-anilino-1-naphthalene sulfonic acid (ANS) molecules, denoted here as 2AN^48^. This molecule has a moderate affinity and low inhibitory potential, but it has been shown that its allosteric binding has positive cooperativity with some orthosteric inhibitors but is relatively noncooperative with ATP^49^. This allosteric site provides interesting clues for selective and/or combinatory treatments of CDK2 in cancer, but almost all cocrystallized ligands are in the orthosteric site, and the underlying assumption made here is that the set of ChEMBL ligands used in affinity prediction bind to this site. Accordingly, the docking box should be defined such that it prevents binding in the 2AN allosteric site to decrease the rate of false positive pose predictions. The same is true for the large binding pocket of the LQ5 ligand, which is a type II inhibitor targeting the inactive DFG-out state of CDK2^44^. A state with the tripeptide DFG motif in outward flip conformation. The final docking box, shown in Fig. 3e, covers both enclosing boxes of ATP/ADP and orthosteric ligands with additional asymmetric padding such that it provides some overlap with 2AN and LQ5 pockets but does not leave enough room for posing ligands in these pockets during docking. It should be noted that receptors with 2AN or LQ5 outside of the orthosteric space (3PXFA and 5A14A) and without any other ligand within it are still members of the nonredundant receptor ensemble (see Table S1). This means that the presence of these ligands out of the docking region induces some unique conformational changes within it.

### Docking without learning

**Figure 4:**
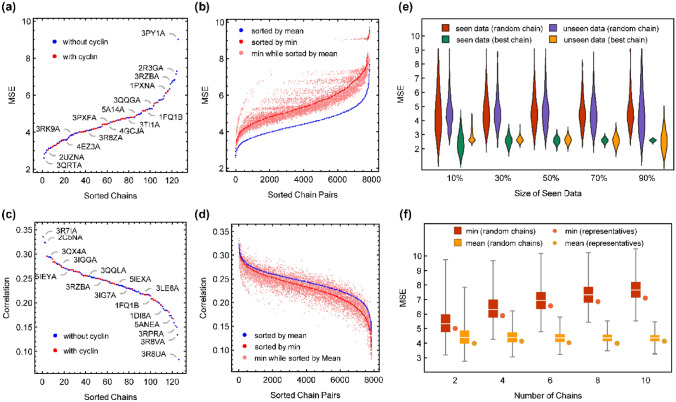
(**a**) MSE of docking predictions of all 126 chains of the non-redundant receptor ensemble. (**b**) MSE of all 7875 ensemble dockings to pairs of receptors. “min” and “mean” refer to using the minimum or average affinities of each ensemble. (**c**,**d**) Same as (**a**) and (**b**) for the Pearson’s correlation coefficient between docking predictions and experimental values. (**e**) Distribution of docking MSE upon selection of best receptor from a random fraction of data (seen data) and using it on the rest of data (unseen data) compared with docking MSE distribution for a randomly selected receptor applied to the same data. (**f**) Docking MSE values of ensembles of different size composed of representative receptors obtained from partitioning of curated receptor ensemble to different numbers of clusters compared to random ensembles of the same size. “min” and “mean” refer to using the minimum or average affinities of each ensemble.

The ligands obtained from ChEMBL for affinity prediction (Table S3) are reasonably diverse, as reflected by their simple molecular properties in Fig. S2 and their reported experimental affinities depicted in Fig. S3. After docking these ligands to all 126 members of the nonredundant receptor ensemble, energetics of the best scored pose for each receptor along with 8 simple molecular properties were used as features (512 in total) for ensemble learning. However, before assessing the benefits of ensemble learning, it is interesting to check the performance of different single receptor or ensemble docking scenarios without any ML rescoring. The MSEs of docking affinity predictions of all 126 receptors are compared in Fig. 4a. The most accurate results were obtained from docking to the 3QRTA receptor (MSE = 2.586), which will be referred to as the **1ChainBestMSE** model. In the same manner, the **1ChainWorstMSE** model is the result of docking to 3PY1A with an MSE of 9.03. The accuracy of any random single chain model would be between these values. Notably, there was no meaningful difference between the docking accuracy of free CDK2 and cyclin-bound CDK2.

The smallest ensemble docking scenario can be made of a pair of receptors. Ensemble docking prediction can be either the average prediction of members (mean scenario) or the best affinity prediction of members (min scenario). As shown in Fig. 4b, the mean scenario has lower MSE values for all possible combinations of receptor pairs. The ensemble {3QRTA, 3R7IA} with MSE values of 2.594 (in the mean scenario) and 2.904 (in the min scenario) is the most accurate ensemble in both scenarios. These two receptors are the most accurate single receptor models (Fig. 4a), and their combination does not provide any advantage in comparison with the single chain results. In other words, there is no 2-chain ensemble docking strategy that performs better than the **1ChainBestMSE** model. The same conclusion can be drawn on the Pearson’s correlation coefficient, as depicted in Figs. 4c and 4d. Both single chain and 2-chain docking predictions show poor correlations with experimental affinities. The **1ChainBestRank** model corresponds to docking to the 3R7IA receptor (${\rho }_{\text{p}}=0.34$ and ${\rho }_{\text{s}}=0.33$), and the **1ChainWorstRank** corresponds to docking to the 3R8UA receptor (${\rho }_{\text{p}}={\rho }_{\text{s}}=0.08$). In the case of 2-chain ensembles, the mean scenario performs slightly better than the min scenario, but for some pairs of receptors, one can find slightly better correlations by using the min of ensemble instead of its mean. Again, none of the 2-chain ensembles can produce correlations better than the **1ChainBestRank** model.

These results were obtained by searching all docking possibilities over whole data. It’s also noteworthy to ask what would be the performance of receptor selection with respect to the enlargement of data. In other words, to what extent the best docking models obtained from a fraction of “seen data” can perform well on the remaining “unseen data”. To address this question, the data were split into different seen/unseen fractions, and the best chain with the lowest docking MSE over the seen data was used to predict unseen data. To compare the performance of this receptor selection strategy with a random situation, the seen/unseen MSE values of a randomly selected receptor were also calculated at each split. The whole procedure was repeated 1000 times, and the corresponding MSE distributions are depicted in Fig. 4e. A random chain model has a constant performance with an average MSE of approximately 4.6 and a large variance. The best selected chain is meaningfully better than random, with an average MSE that decreases slightly from 2.9 to 2.6 over unseen data. The selected best chain seems to be robust, as reflected in the variance of MSE values. With 10% of the data, the most selected receptors were 3QRTA, 3R7IA and 3LE6A in 55.1%, 11.6% and 5.6% of random splits, respectively. Note that these are the three best single chain models in Fig. 4a. The propensity of selecting the 3QRTA receptor increases very fast to 84.3%, 92.7%, 99.4% and 100% by enlarging the seen data to 30%, 50%, 70% and 90% of the whole data, respectively. Accordingly, the **1ChainBestMSE** model is detectable with a high probability by screening of receptors over a small random fraction of whole data.

The last ensemble docking strategy that was assessed is the selection of representative receptors through clustering. From 2 to 10 clusters were considered corresponding to different levels of truncation in Fig. 2a. A representative receptor from each cluster was chosen that has the least dissimilarity to other members of the cluster. Both min and mean scenarios were applied, and the results are compared to random ensembles of the same size in Fig. 4f. In all cases, the ensembles formed by cluster representatives perform slightly better than the random ensembles of the same size. Increasing the size of the ensemble worsens the results of the min scenario for both random ensembles and those formed by cluster representatives. With the mean scenario, the MSE values do not change considerably by enlargement of the ensembles except that the larger random ensembles have lower MSE variance as a result of consensus. It should be noted that the 2VTRA receptor, which is the single representative structure of all 315 receptors (the uppermost arrow in Fig. 2a), has a docking MSE of 3.61 lower than almost all values in Fig. 4f. Accordingly, none of the assessed clustering-based ensemble docking strategies provide superior results, especially if one considers computational cost issues.

### Random forest hyperparameters and trends of error

In the following, our main emphasis is on the RF method since it is easier to tune, more interpretable to select important features, more stable with respect to outliers in the case of small datasets and has provided promising results in the case of protein–ligand interactions^35,50,51^. While there are some rules of thumb for implementing an RF machine, we decided to provide a comprehensive analysis of errors and their trends with respect to hyperparameters, accuracy of models and the results of feature importance rankings.

**Figure 5:**
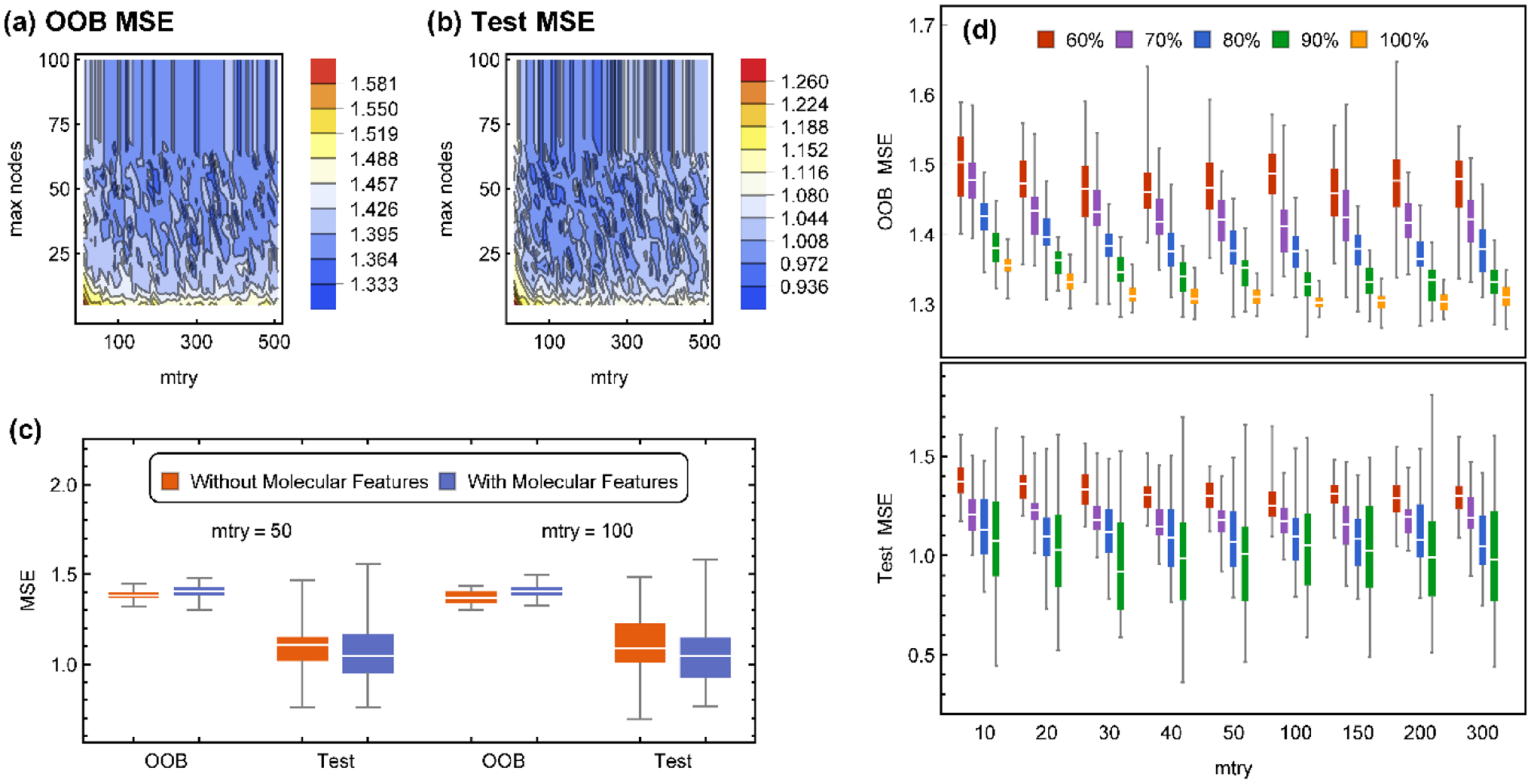
(**a**,**b**) OOB and Test MSE over a grid of different mtry and maxnode values while the number of trees is fixed at 500 and the data is split to 80% train and 20% test. (**c**) The effect of inclusion of molecular features on distribution of OOB and Test MSE obtained upon many random splits of data to 80% train and 20% test. (**d**) Trends of OOB and Test MSE as enlarging the test set from 30 to 100% of data and their dependence on mtry values.

Initial assessments showed that a value of 500 is sufficient for the number of trees (*ntree*), and larger forests have similar trends of sensitivity to other parameters. After a random split of data to 80% train and 20% test sets, the OOB and Test MSE values were calculated over a grid of *mtry* and *maxnode* parameters. The results were depicted in Fig. 5a,b. As a result of randomness, repeating this procedure provides different landscapes of errors except that both errors become independent of the *maxnode* parameter larger than a value of approximately 75. Accordingly, the maximum number of terminal nodes that controls the depth of trees in the forest was set to 80, and only the value of *mtry* was considered an effective hyperparameter in subsequent analyses. The effect of 8 simple molecular features on the distribution of OOB and Test MSE is shown in Fig. 5c for two different values of the *mtry* parameter. While a minor improvement is reflected in this figure for the Test MSE with molecular features, it will be shown that when shrinking the receptor ensemble and reducing the number of receptor-dependent features, the role of these molecular features becomes more prominent.

In Fig. 5d, OOB and Test MSE are depicted for different values of *mtry*. For each value of *mtry,* different fractions of train/test sets were considered to obtain the trend of errors with respect to enlargement of data. Each box plot represents the distribution of errors obtained by 500 random repeats of the whole procedure from data splitting to model building. The enlargement of data successively improves the model accuracy in terms of the median of distributions of both metrics. Enlargement of the train set also decreases the variance of the OOB MSE to a reasonable span. It is well known that the OOB error has a conservative bias in the estimation of the true prediction error^52^. Especially for “small *n*, large *p*” situations, it was shown that the OOB error overestimates the true error depending on the scheme of sampling the variables and the *mtry* value^51^. In the case of the current problem, Fig. 5d shows that this overestimation occurs at all values of *mtry,* but the accuracy of a model built from all data seems to be less sensitive to *mtry* values larger than 50. An *mtry* value of 50 or 100 seems to be a reasonable choice regarding the accuracy of models and keeping the fact in mind that larger values of *mtry* result in more correlated trees and weaken the benefits of consensus in reducing the variance^53^.

### Ranking of receptors via RF feature importance

**Figure 6:**
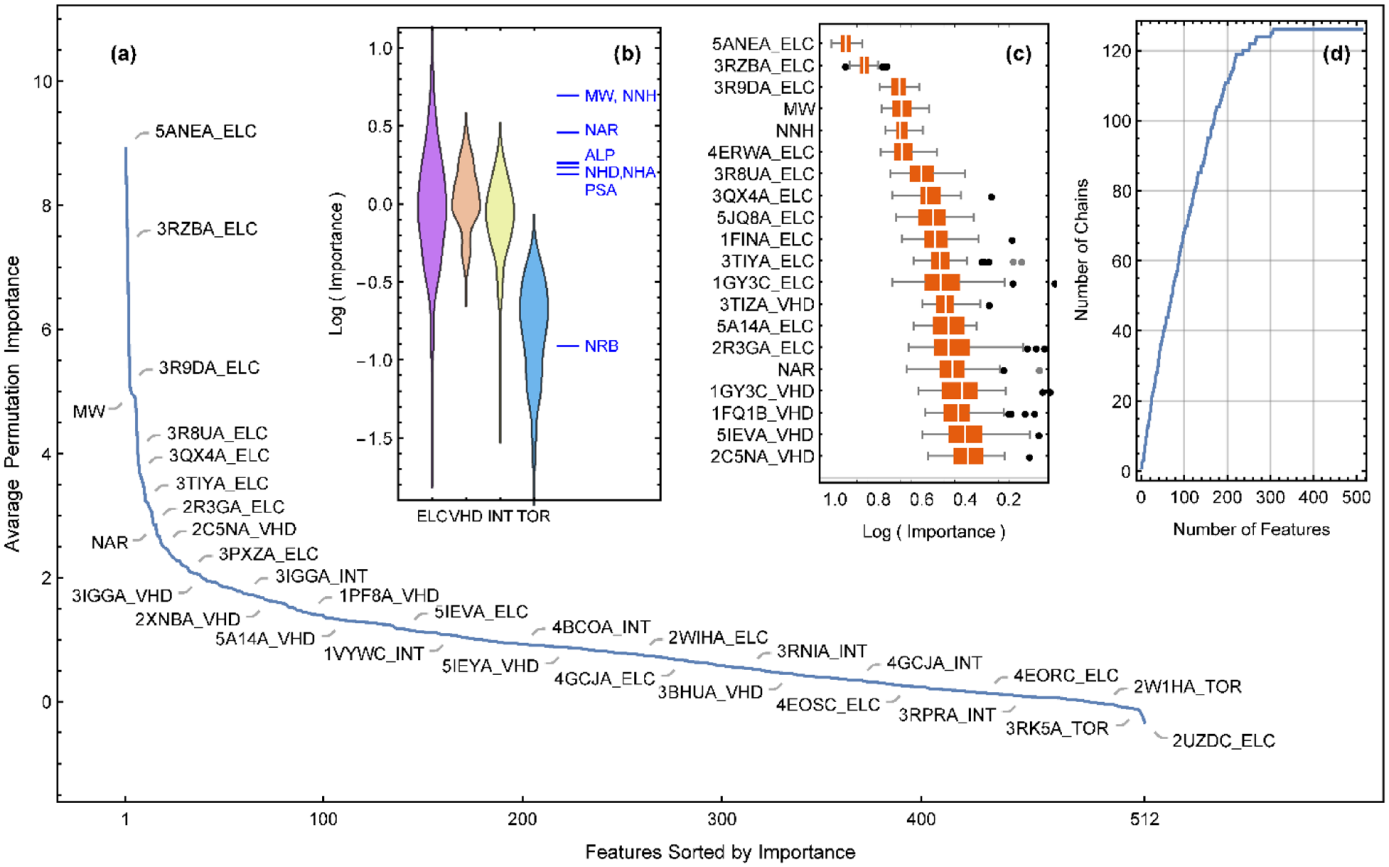
(**a**) Permutation importance of all 512 docking and molecular features averaged over repeats with different random seeds. (**b**) Distribution of average permutation importance of different physical contributions of docking features obtained by aggregating contributions from all receptors. The vertical lines correspond to the average importance of simple molecular features. (**c**) Distribution of permutation importance values of 20 most important features. (**d**) Enumeration rate of receptor chains against features sorted by permutation importance.

The RF model built on the whole set of features seems to be able to reach an average accuracy of 1 kcal/mol in the prediction of binding free energies, but here, the other mission of machine learning is the selection of the most important CDK2 structures for RF-based rescored ensemble docking. The impurity importance that reflects the mean decrease of impurity upon all splittings in the forest is known to be biased when the features vary in their scale of measurements or their number of categories^54^. Some approaches have been suggested to remove this bias, especially in clustering forests^55^. As a result, the permutation importance that reflects the mean decrease in accuracy has been preferred in most feature selection tasks, although it suffers from intensive computational cost for high-dimensional data^54^. Both measures of importance were calculated in this study, and their sensitivity to *mtry* was compared in Fig. S4 for the first 20 important features. Again, the calculations were repeated 500 times to obtain the extent of distributed importance values resulting from the intrinsic randomness of the method. The average permutation importance of all 512 features is plotted in Fig. 6a for *mtry* = 100. Similar results for impurity importance can be found in Fig. S5a. Both importance curves fall rapidly for the first few features, after which the relative preference of successive features becomes less significant regarding the overlap of corresponding box plots (see Figs. S4 and S6c). The magnitude of importance values is sensitive to *mtry,* and especially in the case of impurity importance, an increase in this parameter strengthens the first few important features and weakens the discrimination of features of intermediate importance (see Fig. S4). This can be explained by the fact that by increasing *mtry*, strongly predictive features that are selected in most bootstrap samples have a greater chance of being included in more trees and thus push the measured impurity importance of intermediate features down to the less important ones. However, there is a considerable overlap between the first high ranked features obtained at different *mtry* values or between the impurity and permutation rankings at a specific *mtry*. For both measures, there is 80% overlap between the first 20 important features obtained at four different *mtry* values reported in Fig. S4. At a *mtry* value of 100, which was chosen for subsequent steps of the current work, there is 100%, 80%, 70% and 66% overlap between the impurity and permutation results of the most important 3, 10, 20 and 50 features, respectively. A Spearman’s rank correlation coefficient of 0.76 was found over the whole set of features between impurity and permutation rankings. This increases to approximately 0.9 in the first 20 most important features.

A comparison of the relative importance of different energetic contributions shows that the electrostatic interaction energy (ELC) and the torsional loss of entropy (TOR) are the most and the least important features, respectively (see Figs. 6b and S6b). Both impurity and permutation measures are in agreement on the general importance order of docking contributions: ELC >  > VHD > INT >  > TOR. They are also in agreement about the most important molecular features, the molecular weight (MW) and the number of nonhydrogen atoms (NNH), that are highly correlated representations of nearly the same thing. The least important molecular feature, the number of rotatable bonds (NRB), is consistent with the low importance of torsional loss of entropy (TOR) if one considers the way that the AutoDock force field estimates the latter from the former^9^. Now, the main question is how to rank receptor chains based on feature importance values. Figure 6d shows how fast the number of selected chains can increase when one selects more features in order of their importance. Actually, the relative importance of different physical contributions (Fig. 6b) is such that above a specific cutoff on feature importance, many chains should be selected due to the dominant role played by their ELC or VHD contributions. In practice, the sum of all feature importance values corresponding to a receptor was used to rank all receptors (see Tables S4 and S5).

### Ensemble learning from ensemble docking

**Figure 7:**
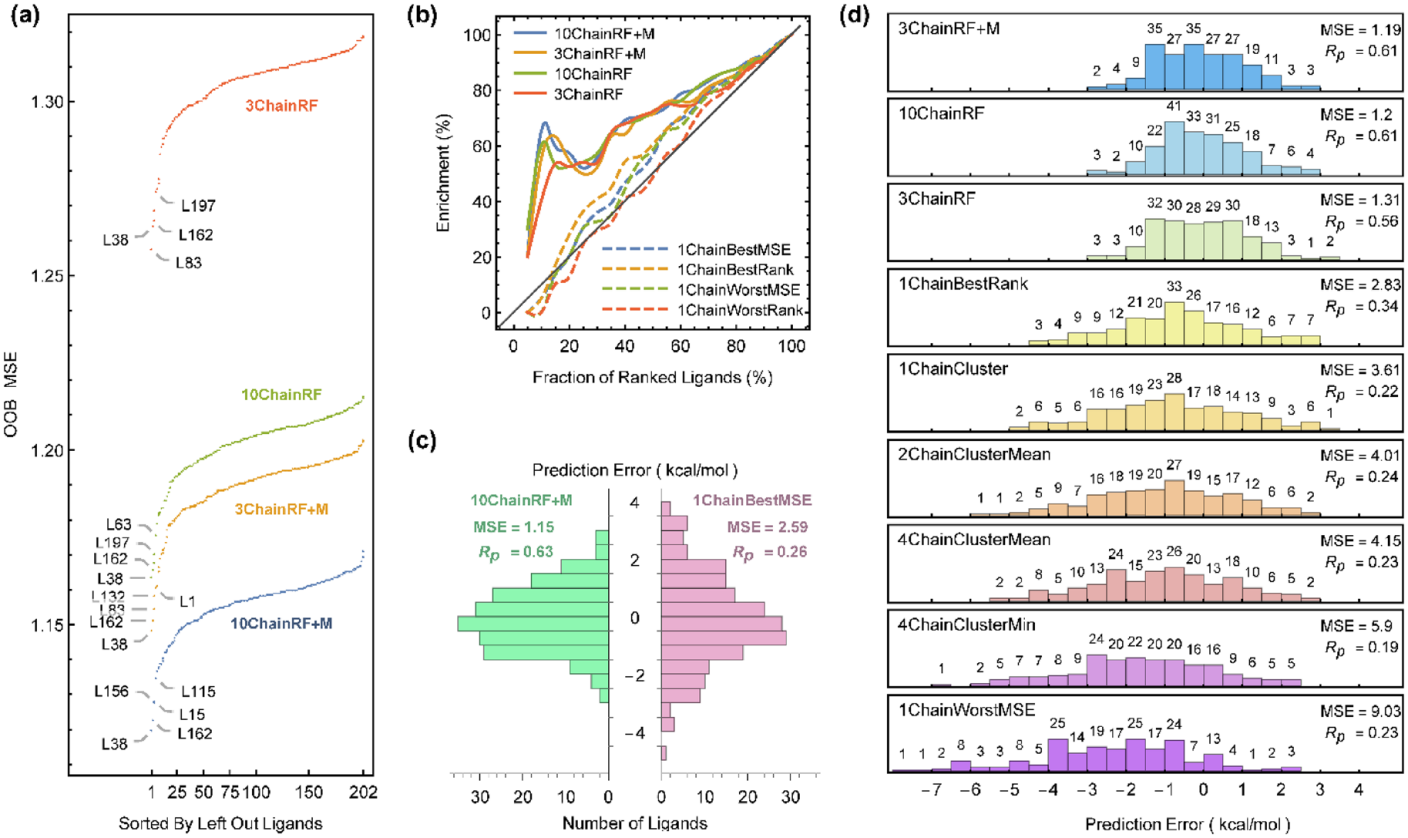
(**a**) OOB MSE for each of the leave-one-out models corresponding to forests created from docking features of 3 and 10 most important receptors with or without 8 molecular features. The most difficult ligands to predict are labeled. (**b**) The enrichment rate of RF models compared to the most and the least accurate docking strategies. (**c**) Distribution of prediction errors of the most accurate RF model in comparison with the most accurate docking without learning strategy. (**d**) Distribution of prediction errors compared for some representative models and their MSE and Pearson’s correlation coefficient between predicted and experimental values.

We compare the results of RF models built from docking features of the 3 and 10 most important receptors, denoted as **3ChainRF**(**+ M**) and **10ChainRF**(**+ M**) models, respectively, where “** + M**” indicates the augmentation of docking features with molecular features. A more deterministic estimate of the model performance was obtained via the calculation of leave-one-out (LOO) errors. This also provides a comparison of per ligand errors between RF models and the single receptor or ensemble docking models discussed above. Single receptor docking, ensemble docking and ensemble learning from docking models were compared with respect to different metrics in Table 1. Figure 7a compares the OOB MSE of all 202 forests that were built for any of the **3ChainRF**, **10ChainRF**, **3ChainRF + M** and **10ChainRF + M** models, which have LOO MSE values of 1.30, 1.20, 1.18 and 1.15, respectively. Some ligands, such as L38 and L162, are commonly difficult to predict in all of these models, and their exclusion reduces the OOB MSE considerably. All different types of MSE and correlation metrics reported thus far measure the performance of models on the complete data. To obtain a sense of performance regarding the early detection problems, the enrichment factor defined in Eq. (6) is plotted in Fig. 7b for the RF models and the best and worst docking results. While the best docking results cannot perform much better than random, the **10ChainRF + M** model can enrich 65% of the top 20% of ranked ligands. Other RF models perform reasonably well, and the existence of simple molecular features (“** + M**” models) enhances early enrichment, especially when one uses only the three most important receptors in ensemble docking. Finally, the distribution of prediction errors was compared between the best docking model (**1ChainBestMSE**) and the best RF model (**10ChainRF + M**), as shown in Fig. 7c. Similar distributions are depicted in Fig. 7d for some of the other models. After docking to the RF selected receptors, RF rescoring not only improves the MSE and correlation but also removes the negative skewness of error distributions, which is a result of false positive predictions in different docking scenarios. Although the main emphasis of the current study was on the RF models, we provided a rough comparison of RF and BRT without any hyperparameter tuning. Figure S6a shows that the BRT model Test MSE decreases with the enlargement of the training data, and Figure S6b compares the LOO errors of the RF and BRT models built on the whole dataset. The performance of both methods seems to be similar, but it is well known that a fine-tuned BRT model can outperform an RF model. On the other hand, such tuning can result in overfitting in the case of noisy data, and we find it safer to prefer RF models regarding the size of available data.

**Table 1:** Different models and their performance measures. In the case of single receptor and ensemble docking models, metrics were obtained from docking predictions, and in the case of RF models, leave-one-out prediction errors were used. EFx% corresponds to the percentage of the top x% of experimentally ranked ligands that correctly predicted in the top x% of computationally ranked ligands.

Models	MSE	MAE	*R*_p_	EF_5%_	EF_20%_	EF_50%_	Receptors
**One receptor docking**							
1ChainBestMSE	2.59	1.27	0.26	0%	20%	56%	3QRTA
1ChainBestRank	2.83	1.36	0.34	0%	27%	60%	3R7IA
1ChainWorstMSE	9.03	3.67	0.23	0%	20%	58%	3PY1A
1ChainWorstRank	5.98	2.02	0.08	0%	12%	48%	3R8UA
**Cluster representatives docking**							
1ChainCluster	3.61	1.55	0.22	0%	12%	57%	2VTRA
2ChainClusterMin	5.01	1.79	0.21	0%	22%	54%	1HCKA, 4EORC
4ChainClusterMin	5.90	1.97	0.19	0%	20%	51%	1HCKA, 3RK9A, 4EORC, 3F5XC
10ChainClusterMin	7.09	2.19	0.24	0%	22%	57%	1HCKA, 3QZHA, 1KE7A, 3RK9A, 3PXFA, 4EOSC, 4EORC, 1VYWC, 1P5EA, 1GY3C
2ChainClusterMean	4.01	1.62	0.24	0%	20%	54%	2VTRA
4ChainClusterMean	4.15	1.66	0.23	0%	22%	53%	1HCKA, 4EORC
10ChainClusterMean	4.11	1.65	0.25	0%	22%	56%	1HCKA, 3RK9A, 4EORC, 3F5XC
**Random forest models**							
3ChainRF	1.31	0.95	0.56	20%	53%	72%	5ANEA, 3RZBA, 4ERWA
10ChainRF	1.20	0.89	0.61	30%	53%	72%	5ANEA, 3RZBA, 4ERWA, 3QX4A, 5JQ8A, 3R8UA, 5A14A, 3R9DA, 1GY3C, 3TIZA
3ChainRF + M	1.19	0.90	0.61	20%	55%	71%	5ANEA, 3RZBA, 4ERWA
10ChainRF + M	1.15	0.88	0.63	20%	58%	71%	5ANEA, 3RZBA, 4ERWA, 3QX4A, 5JQ8A, 3R8UA, 5A14A, 3R9DA, 1GY3C, 3TIZA

## Conclusions

Promising success of machine learning approaches in protein–ligand binding affinity predictions is changing the routines of molecular docking. However, the incorporation of protein flexibility in pipelines of structure-based drug design remains a challenge and makes ensemble docking strategies increasingly popular. On the other hand, the large size of the receptor ensemble affects the performance of the procedure; hence, the optimal size of the receptor ensemble is an open question from both the computational cost and accuracy points of view. In the case of CDK2 and its corresponding available data, we showed that the only meaningful flavor of ensemble docking is its combination with ensemble learning. This outperforms the docking without learning strategies not only with respect to the accuracy, correlation and enrichment metrics but also with regard to efficiency and computational cost concerns. The present results can be seen as a proof of concept for ML-based selection of receptors for subsequent applications in an ensemble docking strategy that should be leveraged by ensemble learning. Four main steps can be suggested: (i) Redundancy removal from an initial set of receptors. (ii) ML-based ranking of receptors over a small but diverse set of ligands. (iii) Ensemble learning from docking to a larger set of ligands. (iv) Application of resulting models to screen large libraries of compounds. This protocol can be extended and improved in different ways. The initial set of receptors can be obtained from computational conformational sampling methods such as different MD-based approaches, especially in the case of proteins whose ligand binding space is not characterized diversely. In cases in which larger sets of protein–ligand binding affinity data are available, other base learners can be tested in the ensemble learning stage, and more sophisticated feature selection strategies can be tuned for selection of the optimum receptor ensemble. Interestingly, we note that while this manuscript was under review a related work was published^56^. That work considers similar idea of combining machine learning and ensemble docking to design receptor-specific binary classifiers to identify active molecules from inactive ones. Promising results have been found over four different proteins that indicates machine learning classifiers significantly outperform traditional consensus strategies. As such, the results of both papers are complementary and highlight new avenues of research.

## Methods

### Initial and curated receptor ensembles

All available 376 X-ray structures containing at least a CDK2 chain (with UniProt ID P24941 or GenBank ID 30,583,821) were downloaded from the RCSB database. Theses PDB files include 481 CDK2 chains. The main emphasis in the current study is on two major conformational states of CDK2: its free form (266 chains) and its complex with cyclin (203 chains). Accordingly, 12 CDK2 chains that are in complex with other proteins were dropped, and our “initial receptor ensemble” includes 469 conformations of CDK2.

In general, the ATP binding site is the main target of most inhibition studies, and 34 chains contain an ATP or ADP molecule at this site. An extended binding region was defined to cover all residues that might affect ligand binding. The “SITE” and “REMARK 800” records in PDB files were used to extract those residues that are in contact with ATP or ADP. This initial binding pocket (27 residues) was then extended to 41 residues by inspecting protein contacts with any valid ligand in all other chains. Chains without any ligand in this extended region were removed from the initial receptor ensemble. Some other chains were also ignored due to missing residues or mutations in the extended binding site. At this stage, the “curated receptor ensemble” included 315 CDK2 chains, as listed in Table S1. Members of the receptor ensembles will be denoted by their PDB and chain IDs (e.g., 1FINA for PDB ID = “1FIN” and chain ID = “A”). Residues in the defined extended binding region are listed in Table S2.

### Receptor refinements and docking box definition

The pattern of missing residues corresponding to disordered segments of the sequence was analyzed, and all missing residues were reconstructed by the loop modeling module in MODELLER^57^. Hydrogen atoms were added to X-ray structures by the Reduce program^58^. Missing atoms were repaired by the psfgen package in VMD 1.9^59^. NAMD 2.10^60^ in conjunction with the CHARMM 27 force field^61^ was used for 20,000 steps of conjugated gradient minimization while restraining all experimentally determined atom positions. All receptors were superimposed from their backbone atoms to provide a common frame for analysis of ligand positions and defining a unique set of coordinates for ligand docking. Before the docking step and to reduce false positive pose predictions, the experimental position of all ligands and their coordinate bounding boxes were analyzed collectively and via a principal component analysis on the centers of geometry of ligands. Accordingly, a docking box size of 22 × 20 × 21 Å^3^ was chosen in a center that provides enough space for pose search in ATP binding site and its nearby but prevents from posing ligands in an allosteric center behind this site.

### Receptor clustering and redundancy removal

With respect to the geometry of residues in the extended binding site, some structures in the curated receptor ensemble are nearly identical. Moreover, the size of this ensemble (315 conformers) is larger than to be practically suitable for an ensemble docking strategy. To analyze the redundancy or proximity of receptors, a dissimilarity tensor ${\varvec{D}}$ with dimensions of 315 × 315 × 41 was defined as

1 $${D}_{ijk}=RMSD({{\varvec{r}}}_{ik},{{\varvec{r}}}_{jk})$$

where ${{\varvec{r}}}_{ik}$ and ${{\varvec{r}}}_{jk}$ are vectors of nonhydrogen atom positions of kth -residue in ith and jth receptors and RMSD stands for root mean squared deviation. Instead of the common task of calculating RMSD for all 41 binding site residues at once, this definition makes it possible to care about different geometries of every single residue when comparing a pair of receptors. The dissimilarity tensor ${\varvec{D}}$ was then converted to a total RMSD proximity measure ${\varvec{T}}$ defined as follows:

2 $${T}_{ij}=\sum_{k=1}^{41}{D}_{ijk}$$

Each element of ${\varvec{T}}$ adds up all pairwise RMSD values of all binding site residues between two receptors. This accumulation of per residue RMSD values provides a better signal-to-noise ratio in comparison with the calculation of RMSD values of all residues at once. Another proximity measure ${\varvec{N}}$ was also defined as follows:

3 $${N}_{ij}=\sum_{k=1}^{41}{\Theta (D}_{ijk}-2)$$

where $\Theta$ is the Heaviside step function. Accordingly, ${N}_{ij}$ is the number of residues that have an RMSD value larger than 2 Å between the ith and jth receptors. In this manner, single residue conformational differences between receptors were counted through a cutoff. Using both ${\varvec{T}}$ and ${\varvec{N}}$ as dissimilarity matrices, members of the curated receptor ensemble were clustered via a hierarchical agglomerative algorithm with Ward linkage to assess different ways of ensemble shrinking and redundancy removal. In this work, a receptor redundancy criterion was defined as ${N}_{ij}=0$. In other words, two receptors are assumed to be identical if none of the 41 residues in the extended binding site have RMSD values larger than 2 Å between them. A graph-based redundancy removal method was suggested and applied that provides better ensemble shrinkage in a less subjective and more robust design. The proximity measure ${\varvec{N}}$ was converted to an adjacency matrix ${\varvec{A}}$ as follows:

4 $${A}_{ij}=(1-{\Theta (N}_{ij})){\delta }_{ij}$$

where $${\delta }_{ij}$$ is the Kronecker delta that keeps $${\varvec{A}}$$ traceless. On the corresponding graph, each node represents a receptor, and each edge between nodes means that those receptors are identical ($${N}_{ij}=0)$$. An iterative procedure selects the node with a maximum degree as a representative nonredundant receptor and deletes its closed neighborhood from the graph. The iteration stops when no edges remain and the remaining nodes are added to the set of nonredundant receptors. In this manner, 126 CDK2 chains were selected that will be denoted as “nonredundant receptor ensemble” (see Table S1).

### Ensemble docking

A set of 630 ligands with available CDK2 inhibition constants (${k}_{i}$) were obtained from the ChEMBL database^62^. Among them, only those records with an equality relation for ${k}_{i}$ values (not inequalities or ranges) were kept. This set was also filtered based on the availability of atom types in the scoring function of the selected docking tool, no rule of five violations, number of rotatable bonds less than 12 and availability of 3D structures in the ZINC database^63^. After all, 202 ligands were selected for docking to all 126 members of the nonredundant receptor ensemble. In the case of different protonation states in the ZINC database, the structure corresponding to pH = 7 was selected. A list of these ligands can be found in Table S3. In a recent study, we compared AutoDock4^9^ and AutoDock Vina^10^ docking tools regarding their pose prediction accuracy based on available X-ray structures of ligand-CDK2 complexes^64^. It was shown that for the top-ranked predicted pose, i.e., the best scored docking geometry, AutoDock4 reproduced 62% of binding geometries with an RMSD less than 2 Å from the experimental geometry, while this value was 37% for AutoDock Vina. Accordingly, AutoDock4 was used in the current study since better pose prediction in the ensemble docking stage can enhance the subsequent ensemble learning results. The docking parameters were chosen to be the same as those used in a previous study^64^. A Lamarckian genetic algorithm with an initial population of 500 was repeated 200 times for each ligand-receptor complex, and the best scored binding mode was selected for subsequent machine learning steps. AutoDock4 scores complexes according to the following equation:

5 $$\Delta G\approx {\Delta \mathrm{G}}_{vdw}+{\Delta G}_{hbond}+{\Delta G}_{elec}+ {\Delta \mathrm{G}}_{desolv}+{\Delta G}_{tor}$$

which is an estimation of binding free energy ($\Delta G$) based on a linear combination of physically interpretable terms^65,66^. ${\Delta \mathrm{G}}_{vdw}$, ${\Delta G}_{hbond}$ and ${\Delta G}_{elec}$ are van der Waals dispersion/repulsion, hydrogen bonding, and electrostatic interactions, respectively. ${\Delta \mathrm{G}}_{desolv}$ is the desolvation potential, and ${\Delta G}_{tor}$ is an estimate of conformational entropy lost upon binding. Values of these terms for selected complexes were used as features in ensemble learning. However, before ensemble learning, the performance of different ensemble docking scenarios was assessed, including *i*) any of 126 nonredundant receptors, *ii*) all possible 7875 receptor pairs, and *iii*) different numbers of representative receptors obtained from clustering of ${N}_{ij}$ or ${T}_{ij}$. In all cases, comparisons were also made with the same number of receptors randomly selected.

### Ensemble learning

For each ligand and each receptor, four features were extracted from the energetics of their complex reported by AutoDock: 1) VHD is the sum of intermolecular contributions from ${\Delta \mathrm{G}}_{vdw}$, ${\Delta G}_{hbond}$ and ${\Delta \mathrm{G}}_{desolv}$ 2) ELC is the intermolecular contribution from ${\Delta \mathrm{G}}_{elec}$ 3) TOR is ${\Delta G}_{tor}$ 4) INT is the ligand internal energy in the bound state. Since the same conformation was assumed for the bound and unbound states of the ligand, this contribution cancels out from the total free energy but differs between 126 complexes of the same ligand; thus, we kept it in the machine learning step as a feature of ligand binding geometry. Accordingly, for each ligand, we have 504 (4 × 126) receptor-dependent features that will be denoted by combining the receptor ID and the feature symbols, e.g., 5ANEA_VHD and 5ANEA_ELC. This set was augmented by eight simple receptor-independent features of the ligands reported in the ChEMBL database, including molecular weight (MW), AlogP estimation of lipophilicity (ALP), polar surface area (PSA), number of hydrogen bond acceptors (NHA), number of hydrogen bond donors (NHD), number of rotatable bonds (NRB), number of aromatic rings (NAR) and number of nonhydrogen (heavy) atoms (NNH).

Using these features, ensemble learning was utilized to predict experimental binding free energies and to shrink nonredundant receptor ensemble, simultaneously. The random forest (RF) as a bagging approach and the boosted regression trees (BRT) as a boosting approach were used via random forest^67,68^ and gbm^69,70^ R packages, respectively. In RF models, the number of decision trees in the ensemble (*ntree*), the number of features randomly sampled at each split (*mtry*) from which the best splitting criterion is selected and the maximum number of terminal nodes that trees in the forest can have (*maxnode*) were dealt as hyperparameters, and their effect on the performance of models was analyzed extensively. Occasionally, the trends of errors were assessed by splitting the data to train/test sets of different sizes. The performance of the models was measured by the out-of-bag mean squared error (OOB MES) or the test set mean squared error (test MSE). Pearson’s correlation coefficient (${\rho }_{\text{p}}$), Spearman’s rank correlation coefficient (${\rho }_{\text{s}}$) and the leave-one-out (LOO) error were used as other evaluation metrics to compare models. For trend finding and comparative purposes, the inherent random nature of data splitting and model learning were treated by repeating corresponding procedures up to 1000 times to obtain a reliable distribution of considered measures. As a metric for the performance of models in the early recognition of more active fractions of experimentally ranked ligands, an enrichment factor was defined as

6 $${EF}_{x\%}=\frac{\left|{L}_{x\%}^{exp}\cap { L}_{x\%}^{calc}\right|}{\left|{L}_{x\%}^{exp}\right|}\times 100$$

where ${L}_{x\%}^{exp}$ or ${L}_{x\%}^{calc}$ are the subsets of the top $x\%$ ligands sorted by their experimental or calculated affinities, respectively. Accordingly, *EF*_20%_ is the percentage of the top 20% of experimentally ranked ligands that are in the top 20% of computationally ranked ligands.

### Feature importance and receptor ensemble shrinkage

The importance of features in the learning process was used to rank CDK2 chains in the nonredundant receptor ensemble and to assess the extent of reducing the number of structures necessary for ensemble docking while maintaining the accuracy of ensemble learning at a reasonable level. Decision tree models benefit from being considerably interpretable. Each node (attribute/feature) in a decision tree is a selected splitting criterion, and attributes at lower tree depths are considered more important features. A random forest, on the other hand, is a set of decision trees that is not interpretable by relying on the forest structure itself. For this reason, several measures have been introduced to quantify the importance of single features in the final RF model based on which a ranking over feature importance in the final model can be achieved^54,55^. One measure, termed “impurity importance”, was defined as the percentage effect of a feature on the decrease in node impurity averaged over all trees. However, it has been shown that this measure can be strongly biased depending on the underlying distribution of feature values and the size of the data^55^. Thus, we mainly focused on the second measure, termed “permuting importance”, defined as the percentage change in prediction error (MSE) when permuting the feature from out-of-bag (OOB) data and averaging over all trees. A scaling of this measure through dividing the average by the standard deviation of per-tree values was considered since it has been suggested to be beneficial in obtaining more stable importance estimations^54^. Both impurity and permutation measures were calculated by the “importance” function of the randomForest package^71^.

## Supplementary Information

Supplementary Information 1.

Supplementary Information 2.

Supplementary Information 3.

Supplementary Information 4.

Supplementary Information 5.

Supplementary Information 6.

Supplementary Information 7.

Supplementary Information 8.

Supplementary Information 9.

Supplementary Information 10.

Supplementary Information 11.

Supplementary Information 12.

## Data Availability

The data underlying this article are available in the article and in its online supplementary materials.

## Abbreviations

SVC: Support vector classifier
CDK2: Cyclin-dependent kinase 2
AD4: Autodock4
RF: Random forest
BRT: Boosted regression trees
MW: Molecular weight
ALP: AlogP estimation of lipophilicity
PSA: Polar surface area (PSA)
NHA: Number of hydrogen bond acceptors
NHD: Number of hydrogen bond donors
NRB: Number of rotatable bonds
NAR: Number of aromatic rings
NNH: Number of nonhydrogen (heavy) atoms
LOO: Leave-one-out
OOB: Out of bag
ANS: 8-Anilino-1-naphthalene sulfonic acid
ntree: Number of trees
ELC: Electrostatic interaction energy
TOR: Torsional loss of entropy
