# Supplementary figures and images for: Ensemble learning from ensemble docking: revisiting the optimum ensemble size problem
Source: Sci Rep. 2022 Jan 10;12:410. doi: 10.1038/s41598-021-04448-5 (PMC8748946; doi:10.1038/s41598-021-04448-5)

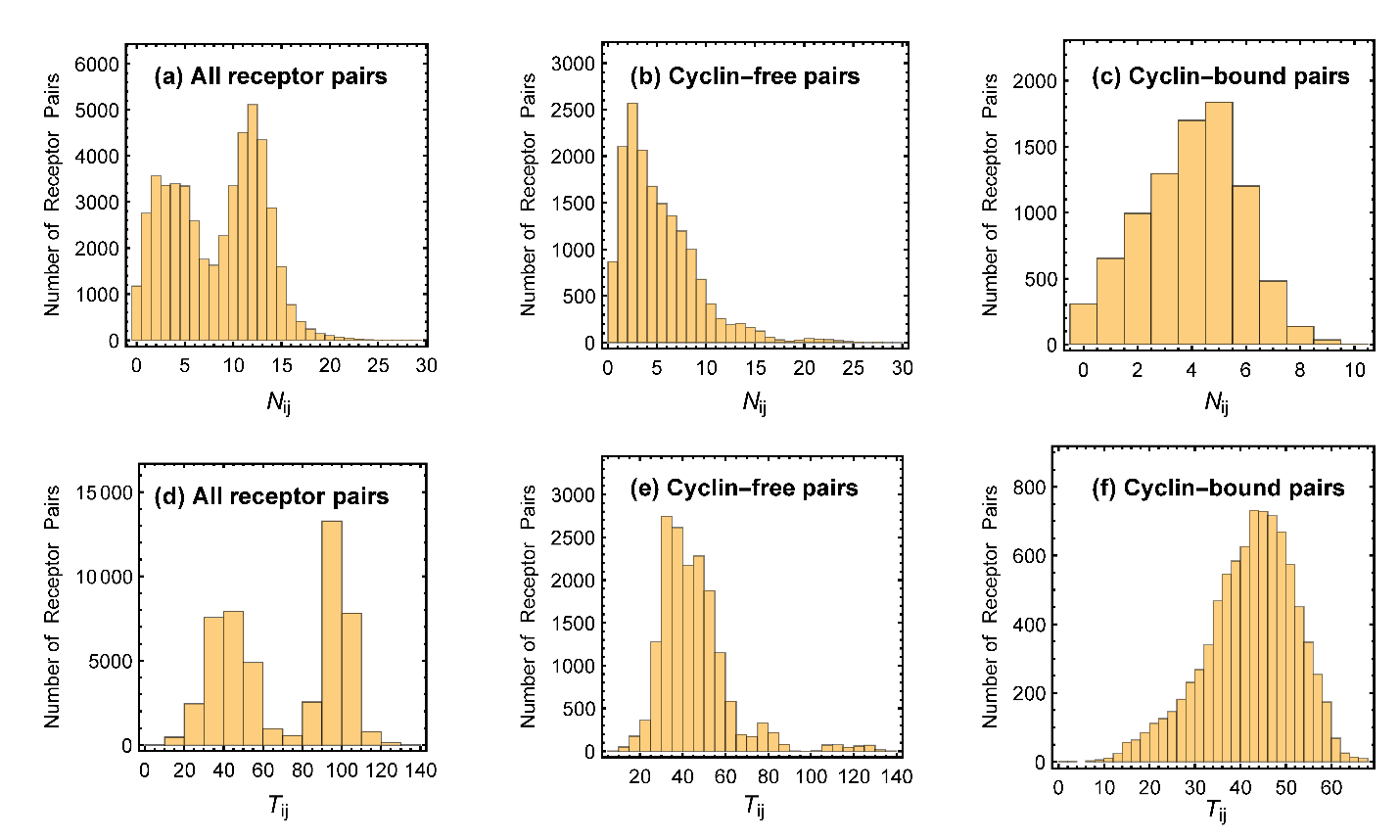


**Figure S1.** Distribution of values of elements in dissimilarity matrices and .

Supplement: Supplementary file 2 — Supplementary Information 2. [file 41598_2021_4448_MOESM2_ESM.docx]
