# Supplementary material for: Ensemble learning from ensemble docking: revisiting the optimum ensemble size problem
Source: Sci Rep. 2022 Jan 10;12:410. doi: 10.1038/s41598-021-04448-5 (PMC8748946; doi:10.1038/s41598-021-04448-5)
Supplement: Supplementary file 3 — Supplementary Information 3. [file 41598_2021_4448_MOESM3_ESM.docx]

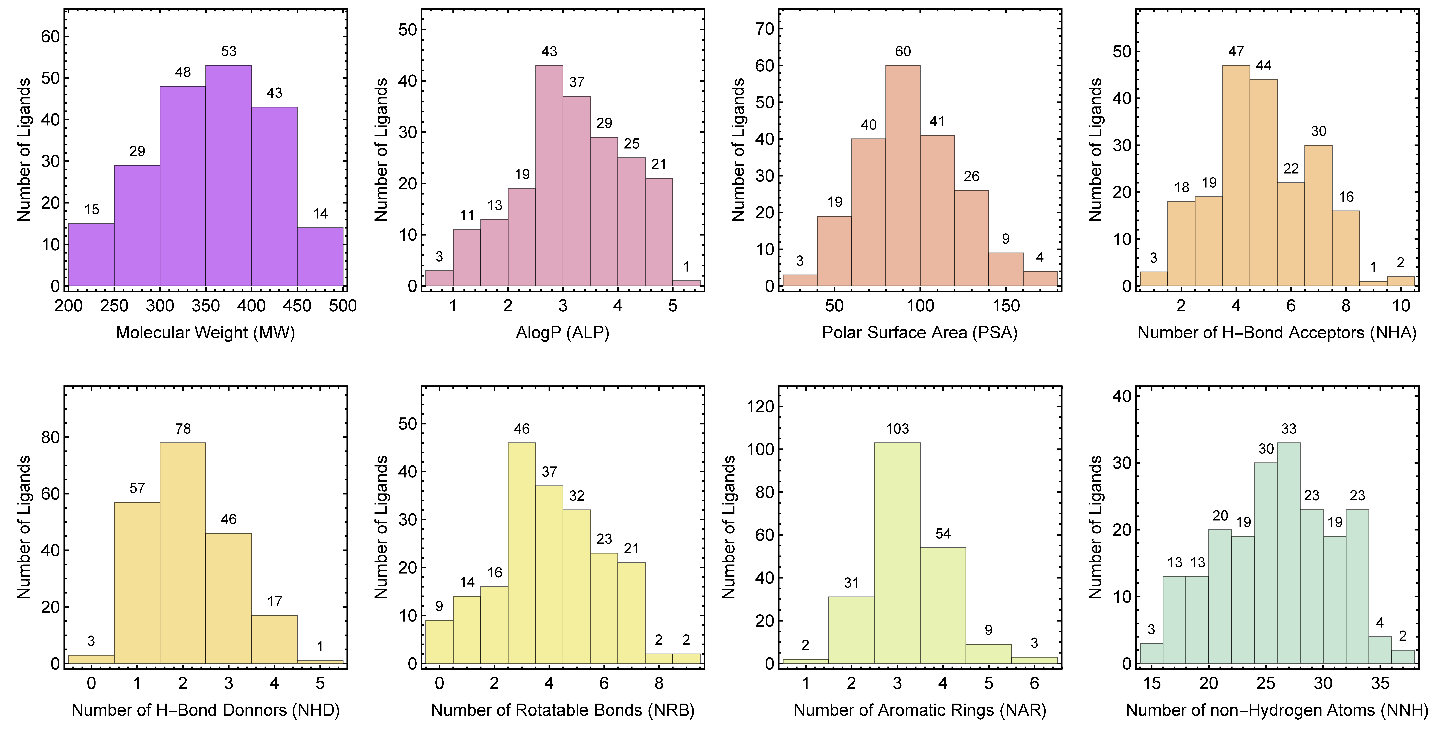


**Figure S2.** Distribution of simple molecular properties for 202 ligands with available CDK2 binding affinity obtained from ChEMBL database.
