# Supplementary material for: Ensemble learning from ensemble docking: revisiting the optimum ensemble size problem
Source: Sci Rep. 2022 Jan 10;12:410. doi: 10.1038/s41598-021-04448-5 (PMC8748946; doi:10.1038/s41598-021-04448-5)
Supplement: Supplementary file 5 — Supplementary Information 5. [file 41598_2021_4448_MOESM5_ESM.docx]

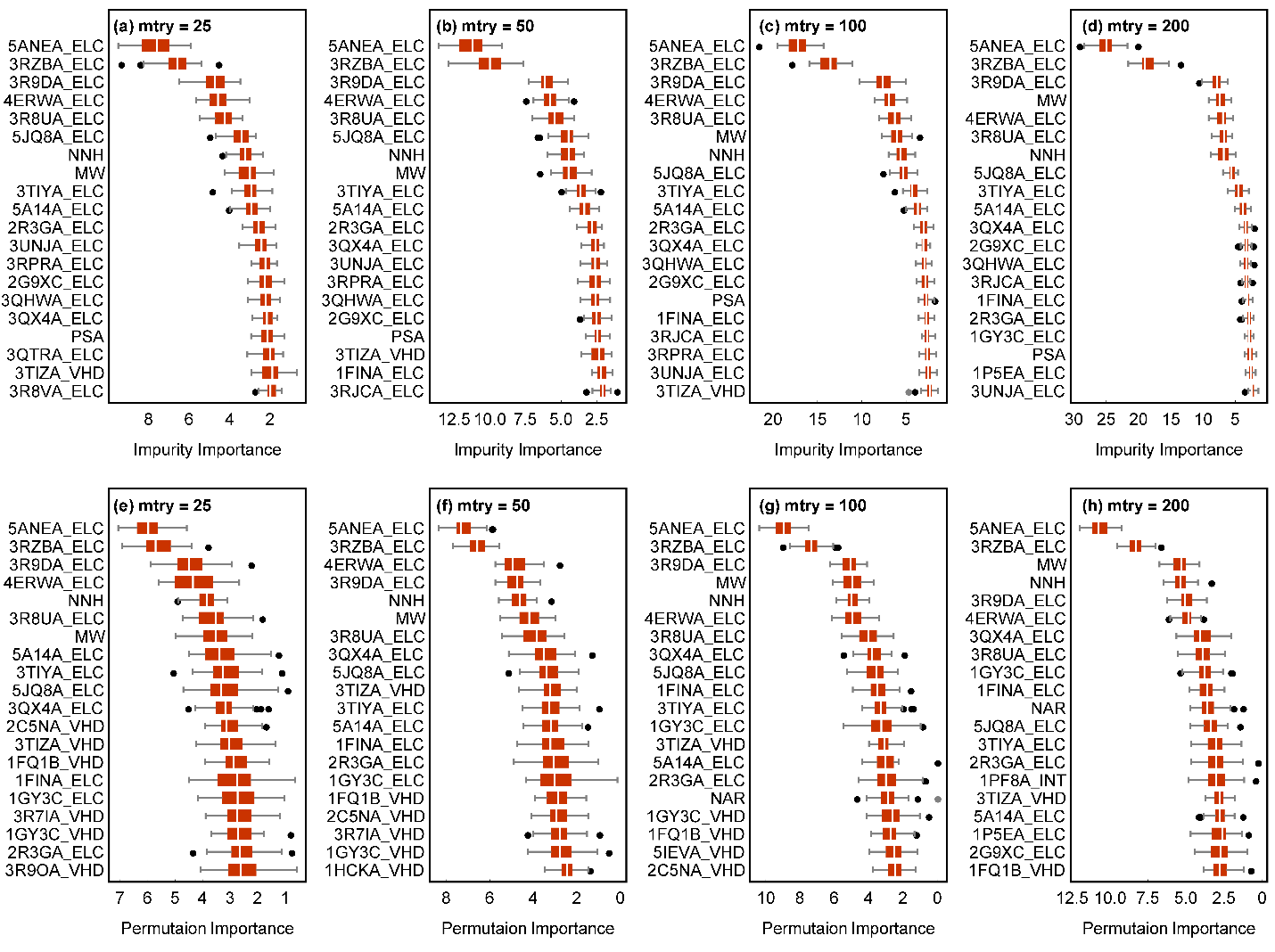


**Figure S4.** Distribution of impurity and permutation importance values for 20 most important features obtained from random repeats of the learning procedure with different values of the *mtry* parameter.
