# Supplementary material for: Ensemble learning from ensemble docking: revisiting the optimum ensemble size problem
Source: Sci Rep. 2022 Jan 10;12:410. doi: 10.1038/s41598-021-04448-5 (PMC8748946; doi:10.1038/s41598-021-04448-5)
Supplement: Supplementary file 6 — Supplementary Information 6. [file 41598_2021_4448_MOESM6_ESM.docx]

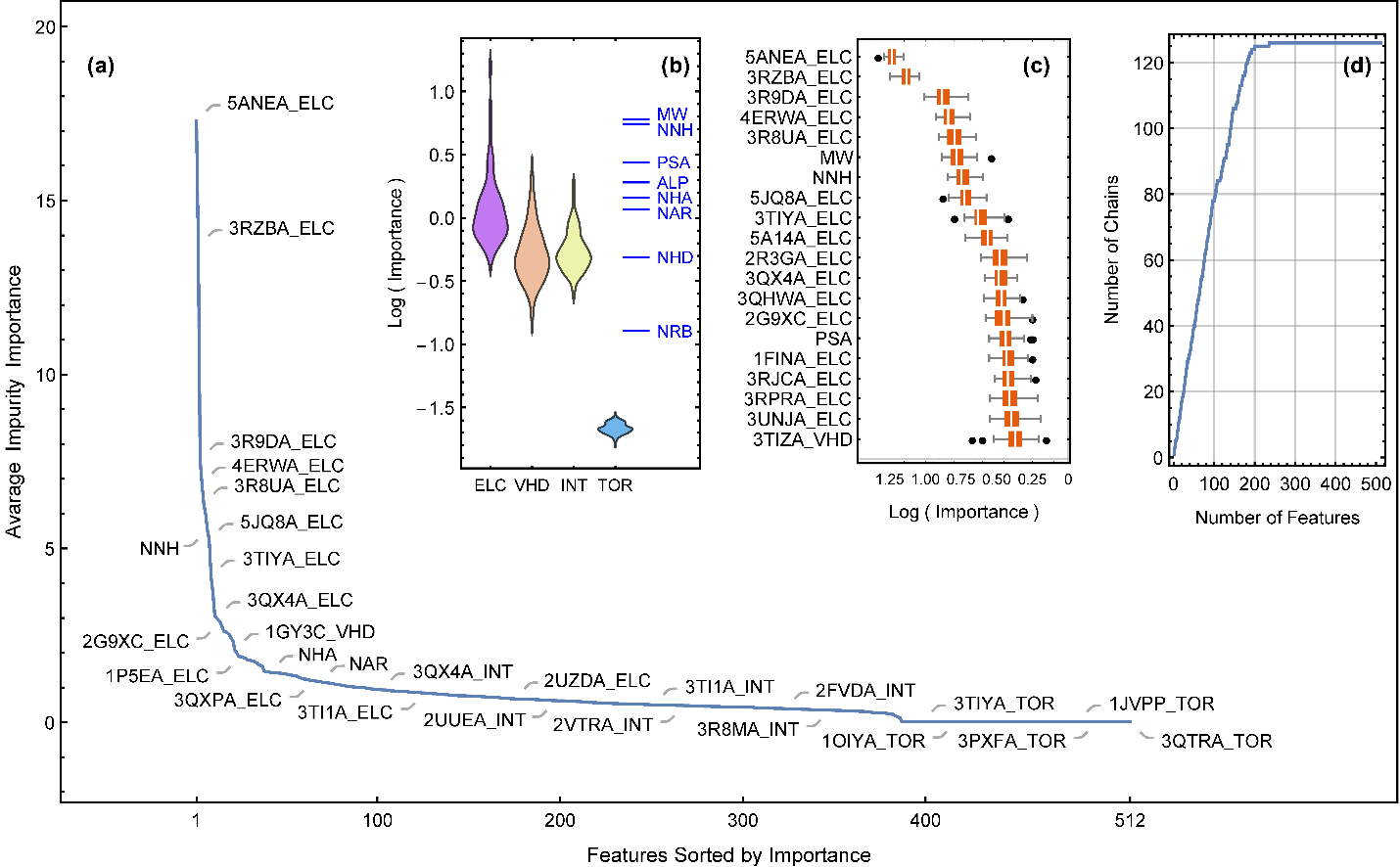


**Figure S5.** (a) Impurity importance of all 512 docking and molecular features averaged over repeats with different random seeds. (b) Distribution of average impurity importance of different physical contributions of docking features obtained by aggregating contributions from all receptors. The vertical lines correspond to the average importance of simple molecular features. (c) Distribution of impurity importance values of 20 most important features. (d) Enumeration rate of receptor chains against features sorted by impurity importance.
