# Supplementary material for: Ensemble learning from ensemble docking: revisiting the optimum ensemble size problem
Source: Sci Rep. 2022 Jan 10;12:410. doi: 10.1038/s41598-021-04448-5 (PMC8748946; doi:10.1038/s41598-021-04448-5)
Supplement: Supplementary file 7 — Supplementary Information 7. [file 41598_2021_4448_MOESM7_ESM.docx]

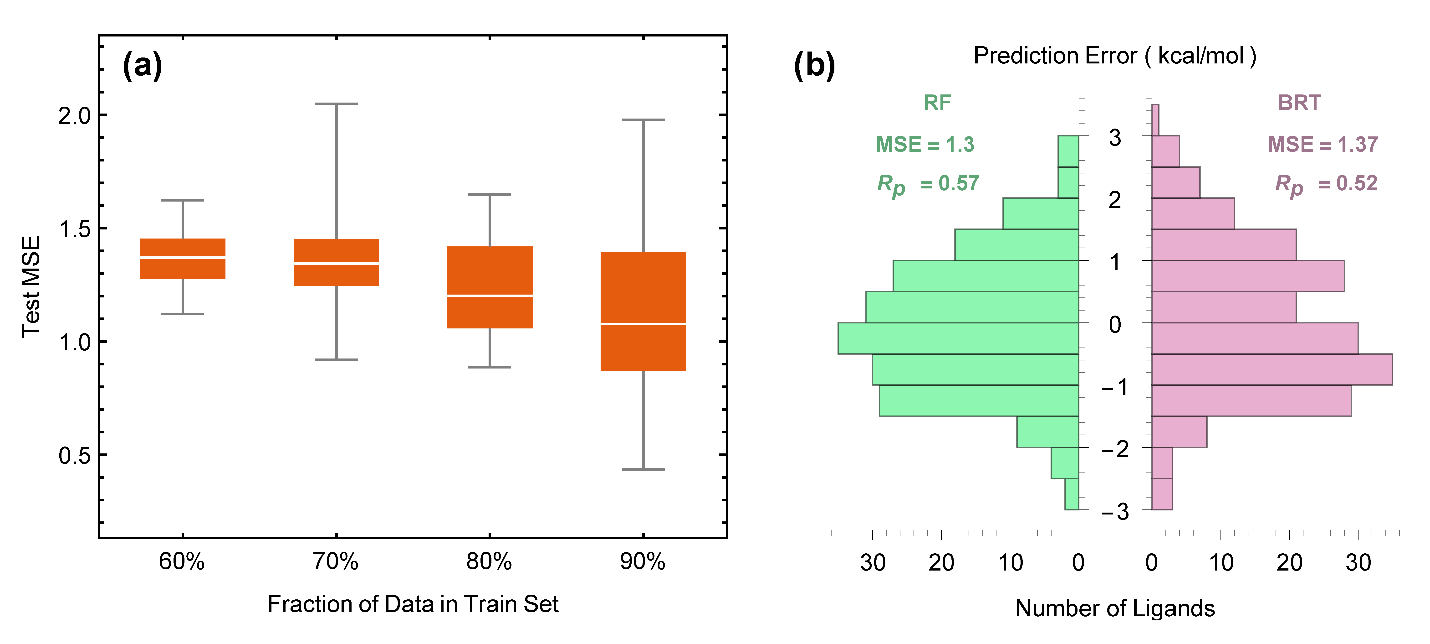


**Figure S6.** (a) Test MSE of boosted regression trees (BRT) method for different fractions of data used as train set. (b) Distribution of leave-one-out errors of random forest (RF) model compared to those obtained from boosted regression trees (BRT) model.
