# Supplementary material for: Ensemble learning from ensemble docking: revisiting the optimum ensemble size problem
Source: Sci Rep. 2022 Jan 10;12:410. doi: 10.1038/s41598-021-04448-5 (PMC8748946; doi:10.1038/s41598-021-04448-5)
Supplement: Supplementary file 8 — Supplementary Information 8. [file 41598_2021_4448_MOESM8_ESM.docx]

**Table S1.** Curated receptor ensemble and its non-redundant subset.

| Receptor | Resolution | Cyclin-Bound | Ligand ID | non-Redundant | Receptor | Resolution | Cyclin-Bound | Ligand ID | non-Redundant | Receptor | Resolution | Cyclin-Bound | Ligand ID | non-Redundant |
| --- | --- | --- | --- | --- | --- | --- | --- | --- | --- | --- | --- | --- | --- | --- |
| 4EK4A | 1.26 | no | 1CK | no | **3ROYA** | 1.75 | no | 22Z | no | **1H0VA** | 1.9 | no | UN4 | no |
| 4FKLA | 1.26 | no | CK2 | no | **3RPOA** | 1.75 | no | 24Z | no | **1HCKA** | 1.9 | no | ATP | yes |
| 2R3IA | 1.28 | no | SCF | no | **3RPRA** | 1.75 | no | 25Z | yes | **2C6KA** | 1.9 | no | DT2 | no |
| 1GZ8A | 1.3 | no | MBP | no | **3NS9A** | 1.78 | no | NS9 | no | **2C6MA** | 1.9 | no | DT5 | no |
| 2R3QA | 1.35 | no | 5SC | no | **1YKRA** | 1.8 | no | 628 | no | **2VTHA** | 1.9 | no | LZ2 | no |
| 4GCJA | 1.42 | no | X64 | yes | **2C6IA** | 1.8 | no | DT1 | no | **2VTSA** | 1.9 | no | LZC | no |
| 2R3RA | 1.47 | no | 6SC | no | **2CLXA** | 1.8 | no | F18 | no | **2XMYA** | 1.9 | no | CDK | no |
| 4FKUA | 1.47 | no | 60K | yes | **2EXMA** | 1.8 | no | ZIP | no | **3FZ1A** | 1.9 | no | B98 | no |
| 2R3FA | 1.5 | no | SC8 | no | **2R3OA** | 1.8 | no | 2SC | no | **3PXQA** | 1.9 | no | 2AN | yes |
| 2R3HA | 1.5 | no | SCE | no | **3IG7A** | 1.8 | no | EFP | yes | **3QL8A** | 1.9 | no | X01 | yes |
| 1JVPP | 1.53 | no | LIG | yes | **3IGGA** | 1.8 | no | EFQ | yes | **3QQGA** | 1.9 | no | X06 | yes |
| 2R3GA | 1.55 | no | SC9 | yes | **3PXFA** | 1.8 | no | 2AN | yes | **3QTSA** | 1.9 | no | X46 | no |
| 4EK5A | 1.6 | no | 03K | no | **3PXYA** | 1.8 | no | JWS | no | **3R7EA** | 1.9 | no | X88 | no |
| 5ANJA | 1.6 | no | ZXC | no | **3QTQA** | 1.8 | no | X35 | no | **3R7YA** | 1.9 | no | Z04 | no |
| 2R3PA | 1.66 | no | 3SC | no | **3R1SA** | 1.8 | no | X73 | no | **3R8LA** | 1.9 | no | Z30 | no |
| 5IEYA | 1.66 | no | 6AE | yes | **3R1YA** | 1.8 | no | X76 | no | **3R8VA** | 1.9 | no | Z62 | yes |
| 3PXZA | 1.7 | no | JWS | yes | **3R8MA** | 1.8 | no | Z19 | yes | **3R9OA** | 1.9 | no | Z71 | yes |
| 3QQJA | 1.7 | no | X11 | no | **3R8PA** | 1.8 | no | Z46 | no | **3RZBA** | 1.9 | no | 02Z | yes |
| 3R73A | 1.7 | no | X87 | no | **3RK7A** | 1.8 | no | 08Z | no | **3UNJA** | 1.9 | no | 0BX | yes |
| 3RAIA | 1.7 | no | X85 | no | **3RPVA** | 1.8 | no | 26Z | no | **5ANIA** | 1.9 | no | ES4 | no |
| 3SW4A | 1.7 | no | 18K | yes | **3S00A** | 1.8 | no | Z60 | no | **5ANKA** | 1.9 | no | RJI | no |
| 4EK8A | 1.7 | no | 16K | no | **3QTUA** | 1.82 | no | X44 | yes | **3QX4A** | 1.92 | no | X4B | yes |
| 4KD1A | 1.7 | no | 1QK | no | **3TIYA** | 1.84 | no | TIY | yes | **5JQ5A** | 1.94 | no | I74 | no |
| 5ANEA | 1.7 | no | SZL | yes | **1E1XA** | 1.85 | no | NW1 | no | **5JQ8A** | 1.94 | no | I73 | yes |
| 3PY0A | 1.75 | no | SU9 | no | **2B54A** | 1.85 | no | D05 | yes | **1E1VA** | 1.95 | no | CMG | no |
| 3QQFA | 1.75 | no | X07 | no | **2FVDA** | 1.85 | no | LIA | yes | **1PXIA** | 1.95 | no | CK1 | no |
| 3QRTA | 1.75 | no | X14 | yes | **2XNBA** | 1.85 | no | Y8L | yes | **2A0CX** | 1.95 | no | CK9 | no |
| 3QWJA | 1.75 | no | X6A | no | **3QQLA** | 1.85 | no | X03 | yes | **2C68A** | 1.95 | no | CT6 | no |
| 3QX2A | 1.75 | no | X63 | no | **3QTRA** | 1.85 | no | X36 | yes | **3QRUA** | 1.95 | no | X19 | yes |
| 3QXOA | 1.75 | no | X65 | no | **3QTWA** | 1.85 | no | X3A | yes | **3QTXA** | 1.95 | no | X43 | no |
| 3QXPA | 1.75 | no | X64 | yes | **3QWKA** | 1.85 | no | X62 | yes | **3QU0A** | 1.95 | no | X40 | yes |
| 3QZGA | 1.75 | no | X67 | no | **3R1QA** | 1.85 | no | X75 | no | **3QZHA** | 1.95 | no | X69 | yes |
| 3QZIA | 1.75 | no | X72 | no | **3R7IA** | 1.85 | no | X9I | yes | **3R7VA** | 1.95 | no | Z02 | no |
| 3R28A | 1.75 | no | XA0 | no | **3R8ZA** | 1.85 | no | Z63 | yes | **3R9DA** | 1.95 | no | X6B | yes |
| 3R6XA | 1.75 | no | X84 | no | **3RJCA** | 1.85 | no | 06Z | yes | **3RNIA** | 1.95 | no | 21Z | yes |
| 3R71A | 1.75 | no | X86 | no | **3RK9A** | 1.85 | no | 09Z | yes | **1JSVA** | 1.96 | no | U55 | no |
| 3R7UA | 1.75 | no | X96 | no | **3RM7A** | 1.85 | no | 19Z | no | **1PXOA** | 1.96 | no | CK7 | no |
| 3R83A | 1.75 | no | Z14 | no | **3QQKA** | 1.86 | no | X02 | no | **3PJ8A** | 1.96 | no | 404 | no |
| 3R9NA | 1.75 | no | Z68 | no | **3QQHA** | 1.87 | no | X0A | yes | **3TI1A** | 1.99 | no | B49 | yes |
| 3RAHA | 1.75 | no | O1Z | no | **2VTRA** | 1.89 | no | LZB | yes | **1B38A** | 2 | no | ATP | no |
| Receptor | Resolution | Cyclin-Bound | Ligand ID | non-Redundant | Receptor | Resolution | Cyclin-Bound | Ligand ID | non-Redundant | Receptor | Resolution | Cyclin-Bound | Ligand ID | non-Redundant |
| 1KE6A | 2 | no | LS2 | no | **1W0XC** | 2.2 | no | OLO | no | **5CYIC** | 2 | yes | 55S | no |
| 1KE7A | 2 | no | LS3 | yes | **1WCCA** | 2.2 | no | CIG | no | **4BCKA** | 2.05 | yes | T3E | no |
| 1KE8A | 2 | no | LS4 | no | **2V0DA** | 2.2 | no | C53 | no | **4BCOA** | 2.05 | yes | T6Q | yes |
| 1KE9A | 2 | no | LS5 | no | **1VYZA** | 2.21 | no | N5B | no | **4BCOC** | 2.05 | yes | T6Q | no |
| 1Y8YA | 2 | no | CT7 | no | **2C5YA** | 2.25 | no | MTW | yes | **4I3ZA** | 2.05 | yes | ADP | yes |
| 2B53A | 2 | no | D23 | no | **2VTMA** | 2.25 | no | LZM | no | **4I3ZC** | 2.05 | yes | ADP | no |
| 2VTAA | 2 | no | LZ1 | no | **1PXJA** | 2.3 | no | CK2 | no | **2UUEA** | 2.06 | yes | MTZ | yes |
| 2VTIA | 2 | no | LZ3 | no | **1PXPA** | 2.3 | no | CK8 | no | **2UUEC** | 2.06 | yes | MTZ | no |
| 2VTLA | 2 | no | LZ5 | no | **2C6LA** | 2.3 | no | DT4 | no | **1H1PA** | 2.1 | yes | CMG | no |
| 3LE6A | 2 | no | 2BZ | yes | **2R64A** | 2.3 | no | 740 | no | **1H1PC** | 2.1 | yes | CMG | no |
| 3QTZA | 2 | no | X42 | no | **2UZNA** | 2.3 | no | C96 | yes | **1OI9A** | 2.1 | yes | N20 | no |
| 3QZFA | 2 | no | X66 | no | **2UZOA** | 2.3 | no | C62 | no | **1OI9C** | 2.1 | yes | N20 | no |
| 3R8UA | 2 | no | Z31 | yes | **3S2PA** | 2.3 | no | PMU | no | **2C5NA** | 2.1 | yes | CK8 | yes |
| 3RK5A | 2 | no | 07Z | yes | **5ANDA** | 2.3 | no | 5JE | no | **2C5NC** | 2.1 | yes | CK8 | no |
| 3RKBA | 2 | no | 12Z | yes | **2A4LA** | 2.4 | no | RRC | no | **2C5OA** | 2.1 | yes | CK2 | no |
| 3ULIA | 2 | no | 1N3 | yes | **3LFSA** | 2.4 | no | A07 | no | **2C5OC** | 2.1 | yes | CK2 | no |
| 3WBLA | 2 | no | PDY | no | **4EZ7A** | 2.49 | no | STU | yes | **3BHVA** | 2.1 | yes | VAR | no |
| 4ERWA | 2 | no | STU | yes | **1PXLA** | 2.5 | no | CK4 | no | **3BHVC** | 2.1 | yes | VAR | no |
| 4EZ3A | 2 | no | 0S0 | yes | **1PXNA** | 2.5 | no | CK6 | yes | **3MY5A** | 2.1 | yes | RFZ | no |
| 4LYNA | 2 | no | 1YG | no | **1PF8A** | 2.51 | no | SU9 | yes | **3MY5C** | 2.1 | yes | RFZ | no |
| 5A14A | 2 | no | LQ5 | yes | **1PXMA** | 2.53 | no | CK5 | yes | **4BCNA** | 2.1 | yes | T9N | no |
| 3TIZA | 2.02 | no | 3TI | yes | **1PXKA** | 2.8 | no | CK3 | yes | **4BCNC** | 2.1 | yes | T9N | yes |
| 3LFQA | 2.03 | no | A28 | no | **1FQ1B** | 3 | no | ATP | yes | **4EOQA** | 2.15 | yes | ATP | yes |
| 5IEVA | 2.03 | no | R0N | yes | **2CCHA** | 1.7 | yes | ATP | yes | **4EOQC** | 2.15 | yes | ATP | no |
| 5IEXA | 2.03 | no | 6AF | yes | **2CCHC** | 1.7 | yes | ATP | yes | **4II5A** | 2.15 | yes | ADP | no |
| 1CKPA | 2.05 | no | PVB | yes | **3DDQA** | 1.8 | yes | RRC | no | **4II5C** | 2.15 | yes | ADP | yes |
| 1W8CA | 2.05 | no | N69 | no | **3DDQC** | 1.8 | yes | RRC | no | **3QHRA** | 2.17 | yes | ADP | no |
| 3PY1A | 2.05 | no | SU9 | yes | **3QHWA** | 1.91 | yes | ADP | yes | **3QHRC** | 2.17 | yes | ADP | no |
| 1B39A | 2.1 | no | ATP | no | **3QHWC** | 1.91 | yes | ADP | no | **1QMZA** | 2.2 | yes | ATP | no |
| 1H0WA | 2.1 | no | 207 | no | **1H1RA** | 2 | yes | 6CP | no | **1QMZC** | 2.2 | yes | ATP | no |
| 2C69A | 2.1 | no | CT8 | no | **1H1RC** | 2 | yes | 6CP | no | **4CFNA** | 2.2 | yes | JYM | no |
| 2C6OA | 2.1 | no | 4SP | no | **1H1SA** | 2 | yes | 4SP | no | **4CFNC** | 2.2 | yes | JYM | no |
| 3R9HA | 2.1 | no | Z67 | no | **1H1SC** | 2 | yes | 4SP | no | **4CFUA** | 2.2 | yes | 2WC | no |
| 3UNKA | 2.1 | no | 0BY | no | **1OIUA** | 2 | yes | N76 | yes | **4CFUC** | 2.2 | yes | 2WC | yes |
| 1Y91A | 2.15 | no | CT9 | yes | **1OIUC** | 2 | yes | N76 | no | **4EORA** | 2.2 | yes | 4SP | yes |
| 2VTPA | 2.15 | no | LZ9 | no | **3BHTA** | 2 | yes | MFR | yes | **4EORC** | 2.2 | yes | 4SP | yes |
| 2W1HA | 2.15 | no | L0F | yes | **3BHTC** | 2 | yes | MFR | no | **1P5EA** | 2.22 | yes | TBS | yes |
| 2VTOA | 2.19 | no | LZ8 | no | **3TNWA** | 2 | yes | F18 | yes | **1P5EC** | 2.22 | yes | TBS | yes |
| 1DI8A | 2.2 | no | DTQ | yes | **4CFVA** | 2 | yes | 75X | yes | **4BCPA** | 2.26 | yes | T3C | no |
| 1FVTA | 2.2 | no | 106 | no | **5CYIA** | 2 | yes | 55S | no | **1FINA** | 2.3 | yes | ATP | yes |
