# Supplementary material for: Ensemble learning from ensemble docking: revisiting the optimum ensemble size problem
Source: Sci Rep. 2022 Jan 10;12:410. doi: 10.1038/s41598-021-04448-5 (PMC8748946; doi:10.1038/s41598-021-04448-5)
Supplement: Supplementary file 9 — Supplementary Information 9. [file 41598_2021_4448_MOESM9_ESM.docx]

**Table S2.** Extended binding region residues and their number of contacts with 320 ligands in 315 CDK2 chains.

| Residue | | Number of  Ligand Contacts |
| --- | --- | --- |
| Name | **ID** |  |
| Leu | 83 | 313 |
| Ala | 31 | 304 |
| Leu | 134 | 303 |
| Glu | 81 | 301 |
| Ile | 10 | 253 |
| His | 84 | 229 |
| Phe | 80 | 202 |
| Phe | 82 | 200 |
| Asp | 86 | 189 |
| Lys | 33 | 172 |
| Asp | 145 | 170 |
| Lys | 89 | 144 |
| Val | 18 | 135 |
| Gln | 85 | 129 |
| Gln | 131 | 119 |
| Asn | 132 | 110 |
| Glu | 12 | 85 |
| Val | 64 | 82 |
| Gly | 13 | 75 |
| Gly | 11 | 49 |
| Ala | 144 | 42 |
| Tyr | 15 | 19 |
| Glu | 8 | 18 |
| Thr | 14 | 14 |
| Glu | 51 | 11 |
| Lys | 129 | 10 |
| Gly | 16 | 7 |
| Phe | 146 | 5 |
| Leu | 55 | 4 |
| Asp | 127 | 4 |
| Lys | 9 | 3 |
| Leu | 148 | 3 |
| Leu | 78 | 3 |
| Phe | 152 | 2 |
| Gly | 147 | 2 |
| Val | 123 | 1 |
| Leu | 143 | 1 |
| Leu | 124 | 1 |
| Leu | 58 | 1 |
| Ile | 63 | 1 |
| His | 125 | 1 |
