# Supplementary material for: Ensemble learning from ensemble docking: revisiting the optimum ensemble size problem
Source: Sci Rep. 2022 Jan 10;12:410. doi: 10.1038/s41598-021-04448-5 (PMC8748946; doi:10.1038/s41598-021-04448-5)
Supplement: Supplementary file 10 — Supplementary Information 10. [file 41598_2021_4448_MOESM10_ESM.docx]

**Table S3.** Ligands, their database IDs and reported CDK2 inhibition constants.

| Ligand | ChEMBL ID | ZINC ID | Ki | Ligand | ChEMBL ID | ZINC ID | Ki |
| --- | --- | --- | --- | --- | --- | --- | --- |
| L1 | CHEMBL1076721 | ZINC000049525584 | 62 | **L41** | CHEMBL1969473 | ZINC000063539987 | 63.1 |
| L2 | CHEMBL1080902 | ZINC000049033769 | 95 | **L42** | CHEMBL1969561 | ZINC000063298297 | 100 |
| L3 | CHEMBL1083484 | ZINC000036403407 | 3967 | **L43** | CHEMBL1969664 | ZINC000038698888 | 1000 |
| L4 | CHEMBL1083786 | ZINC000034105115 | 432 | **L44** | CHEMBL1969843 | ZINC000063298300 | 1995.26 |
| L5 | CHEMBL1084630 | ZINC000003950136 | 151 | **L45** | CHEMBL1971245 | ZINC000063298250 | 7943.28 |
| L6 | CHEMBL116070 | ZINC000000026008 | 1000 | **L46** | CHEMBL1971801 | ZINC000038762686 | 2511.89 |
| L7 | CHEMBL1190711 | ZINC000003817793 | 316.23 | **L47** | CHEMBL1971943 | ZINC000043201623 | 398.11 |
| L8 | CHEMBL1241473 | ZINC000003986637 | 15.85 | **L48** | CHEMBL1972158 | ZINC000063298257 | 50.12 |
| L9 | CHEMBL1487428 | ZINC000001452379 | 19952.62 | **L49** | CHEMBL1972362 | ZINC000063298476 | 1000 |
| L10 | CHEMBL1562756 | ZINC000100640971 | 6309.57 | **L50** | CHEMBL1973893 | ZINC000063298504 | 100 |
| L11 | CHEMBL1682357 | ZINC000063298308 | 794.33 | **L51** | CHEMBL1975787 | ZINC000063298474 | 251.19 |
| L12 | CHEMBL1682359 | ZINC000063298315 | 630.96 | **L52** | CHEMBL1975937 | ZINC000063540148 | 6309.57 |
| L13 | CHEMBL1682540 | ZINC000063298311 | 1258.93 | **L53** | CHEMBL1976134 | ZINC000063298526 | 1258.93 |
| L14 | CHEMBL1682553 | ZINC000063298288 | 251.19 | **L54** | CHEMBL1976420 | ZINC000063298517 | 251.19 |
| L15 | CHEMBL1725279 | ZINC000100706870 | 79.43 | **L55** | CHEMBL1977346 | ZINC000063298570 | 316.23 |
| L16 | CHEMBL178737 | ZINC000003817328 | 316.23 | **L56** | CHEMBL1980391 | ZINC000035930738 | 158.49 |
| L17 | CHEMBL184510 | ZINC000013582603 | 1 | **L57** | CHEMBL1982271 | ZINC000063539726 | 39.81 |
| L18 | CHEMBL185099 | ZINC000014945958 | 270 | **L58** | CHEMBL1982660 | ZINC000063298411 | 100 |
| L19 | CHEMBL185310 | ZINC000014945953 | 90 | **L59** | CHEMBL1983309 | ZINC000005358225 | 3162.28 |
| L20 | CHEMBL186288 | ZINC000013582625 | 20 | **L60** | CHEMBL1983589 | ZINC000063539782 | 398.11 |
| L21 | CHEMBL186708 | ZINC000012355037 | 6 | **L61** | CHEMBL1983932 | ZINC000063298417 | 794.33 |
| L22 | CHEMBL187081 | ZINC000013582569 | 2 | **L62** | CHEMBL1984363 | ZINC000063298467 | 3981.07 |
| L23 | CHEMBL188303 | ZINC000013582641 | 190 | **L63** | CHEMBL1984548 | ZINC000063540050 | 1.259 |
| L24 | CHEMBL188312 | ZINC000013582619 | 60 | **L64** | CHEMBL1985074 | ZINC000063298539 | 7943.28 |
| L25 | CHEMBL188417 | ZINC000013582627 | 190 | **L65** | CHEMBL1985095 | ZINC000063298087 | 398.11 |
| L26 | CHEMBL188665 | ZINC000013582596 | 10 | **L66** | CHEMBL1985723 | ZINC000063539659 | 501.19 |
| L27 | CHEMBL189235 | ZINC000013582613 | 160 | **L67** | CHEMBL1986499 | ZINC000043208592 | 125.89 |
| L28 | CHEMBL189455 | ZINC000012354947 | 10 | **L68** | CHEMBL1986943 | ZINC000063298130 | 12.59 |
| L29 | CHEMBL1964307 | ZINC000038318181 | 2511.89 | **L69** | CHEMBL1987034 | ZINC000034605225 | 7.943 |
| L30 | CHEMBL1964340 | ZINC000063539783 | 251.19 | **L70** | CHEMBL1988163 | ZINC000063298121 | 25.12 |
| L31 | CHEMBL1964444 | ZINC000063298413 | 79.43 | **L71** | CHEMBL1988537 | ZINC000063298592 | 1995.26 |
| L32 | CHEMBL1964644 | ZINC000003819045 | 2511.89 | **L72** | CHEMBL1988838 | ZINC000063298077 | 199.53 |
| L33 | CHEMBL1964804 | ZINC000063298271 | 398.11 | **L73** | CHEMBL1989069 | ZINC000063539566 | 19952.62 |
| L34 | CHEMBL1965131 | ZINC000063298388 | 1995.26 | **L74** | CHEMBL1989136 | ZINC000063540014 | 1000 |
| L35 | CHEMBL1965660 | ZINC000063298447 | 63.1 | **L75** | CHEMBL1990212 | ZINC000063540338 | 6309.57 |
| L36 | CHEMBL1965988 | ZINC000063298198 | 2511.89 | **L76** | CHEMBL1992231 | ZINC000063540053 | 1000 |
| L37 | CHEMBL1966425 | ZINC000063540087 | 1258.93 | **L77** | CHEMBL1992363 | ZINC000063539996 | 50.12 |
| L38 | CHEMBL1967544 | ZINC000038749822 | 7943.28 | **L78** | CHEMBL1992644 | ZINC000001502385 | 12589.25 |
| L39 | CHEMBL1967704 | ZINC000063539945 | 316.23 | **L79** | CHEMBL1992740 | ZINC000063298561 | 1000 |
| L40 | CHEMBL1969042 | ZINC000063298587 | 1258.93 | **L80** | CHEMBL1993648 | ZINC000063539743 | 1258.93 |

| Ligand | ChEMBL ID | ZINC ID | Ki | Ligand | ChEMBL ID | ZINC ID | Ki |
| --- | --- | --- | --- | --- | --- | --- | --- |
| L81 | CHEMBL1994074 | ZINC000010150751 | 6309.57 | **L121** | CHEMBL2348643 | ZINC000095603258 | 26 |
| L82 | CHEMBL1994538 | ZINC000043195060 | 1000 | **L122** | CHEMBL2348646 | ZINC000095604947 | 42 |
| L83 | CHEMBL1994669 | ZINC000063540041 | 1.585 | **L123** | CHEMBL2348647 | ZINC000095602927 | 20 |
| L84 | CHEMBL1995592 | ZINC000063298113 | 794.33 | **L124** | CHEMBL2348652 | ZINC000095603580 | 320 |
| L85 | CHEMBL1995740 | ZINC000038495812 | 63.1 | **L125** | CHEMBL2348841 | ZINC000095602434 | 226 |
| L86 | CHEMBL1995811 | ZINC000063539965 | 25.12 | **L126** | CHEMBL2348842 | ZINC000095603317 | 2 |
| L87 | CHEMBL1996111 | ZINC000063298302 | 199.53 | **L127** | CHEMBL2348843 | ZINC000095601088 | 1.5 |
| L88 | CHEMBL1996390 | ZINC000063298478 | 7943.28 | **L128** | CHEMBL243088 | ZINC000014960274 | 316.23 |
| L89 | CHEMBL1996979 | ZINC000063298106 | 3981.07 | **L129** | CHEMBL243298 | ZINC000014960288 | 316.23 |
| L90 | CHEMBL1997597 | ZINC000063298280 | 3981.07 | **L130** | CHEMBL248757 | ZINC000028963230 | 3162.28 |
| L91 | CHEMBL1998193 | ZINC000004015766 | 6309.57 | **L131** | CHEMBL249697 | ZINC000001554390 | 1995.26 |
| L92 | CHEMBL1999918 | ZINC000063298473 | 316.23 | **L132** | CHEMBL259850 | ZINC000003818778 | 5011.87 |
| L93 | CHEMBL2000071 | ZINC000063298173 | 630.96 | **L133** | CHEMBL260135 | ZINC000005224686 | 1584.89 |
| L94 | CHEMBL2000104 | ZINC000063298481 | 1584.89 | **L134** | CHEMBL260933 | ZINC000029055563 | 5011.87 |
| L95 | CHEMBL2000335 | ZINC000063298195 | 7943.28 | **L135** | CHEMBL269881 | ZINC000003873285 | 12000 |
| L96 | CHEMBL2001239 | ZINC000063298107 | 3162.28 | **L136** | CHEMBL280074 | ZINC000001641925 | 3981.07 |
| L97 | CHEMBL2002322 | ZINC000063298477 | 3162.28 | **L137** | CHEMBL296468 | ZINC000003816409 | 38 |
| L98 | CHEMBL2003271 | ZINC000063298358 | 794.33 | **L138** | CHEMBL296586 | ZINC000001639355 | 100 |
| L99 | CHEMBL2003524 | ZINC000000187946 | 398.11 | **L139** | CHEMBL297412 | ZINC000013559864 | 40 |
| L100 | CHEMBL2003637 | ZINC000003818004 | 630.96 | **L140** | CHEMBL298445 | ZINC000002047389 | 2 |
| L101 | CHEMBL2005528 | ZINC000063298568 | 5011.87 | **L141** | CHEMBL302449 | ZINC000001554668 | 15.85 |
| L102 | CHEMBL2005828 | ZINC000063298214 | 7943.28 | **L142** | CHEMBL303958 | ZINC000003814479 | 1300 |
| L103 | CHEMBL2006156 | ZINC000033636836 | 3162.28 | **L143** | CHEMBL3087431 | ZINC000043101526 | 294 |
| L104 | CHEMBL2007002 | ZINC000063298378 | 794.33 | **L144** | CHEMBL336961 | ZINC000013588927 | 501.19 |
| L105 | CHEMBL2064558 | ZINC000084726009 | 1800 | **L145** | CHEMBL340384 | ZINC000027306749 | 251.19 |
| L106 | CHEMBL2064561 | ZINC000084727551 | 2000 | **L146** | CHEMBL359999 | ZINC000013582599 | 10 |
| L107 | CHEMBL2064562 | ZINC000043170004 | 1160 | **L147** | CHEMBL360408 | ZINC000013582654 | 3980 |
| L108 | CHEMBL210618 | ZINC000014959960 | 794.33 | **L148** | CHEMBL361697 | ZINC000013582606 | 310 |
| L109 | CHEMBL213713 | ZINC000014950281 | 2 | **L149** | CHEMBL361894 | ZINC000012354995 | 6 |
| L110 | CHEMBL214183 | ZINC000003963103 | 495 | **L150** | CHEMBL362722 | ZINC000014945955 | 80 |
| L111 | CHEMBL215086 | ZINC000014950278 | 6 | **L151** | CHEMBL3632736 | ZINC000000450245 | 3046 |
| L112 | CHEMBL215205 | ZINC000100035856 | 69000 | **L152** | CHEMBL364408 | ZINC000014945947 | 50 |
| L113 | CHEMBL219722 | ZINC000036176941 | 6309.57 | **L153** | CHEMBL364767 | ZINC000014945956 | 30 |
| L114 | CHEMBL223367 | ZINC000014958346 | 5760 | **L154** | CHEMBL365229 | ZINC000013582601 | 10 |
| L115 | CHEMBL225519 | ZINC000016052718 | 316.23 | **L155** | CHEMBL365617 | ZINC000004617893 | 6309.57 |
| L116 | CHEMBL226232 | ZINC000028645388 | 1995.26 | **L156** | CHEMBL375530 | ZINC000003873418 | 65000 |
| L117 | CHEMBL2312189 | ZINC000043207877 | 123 | **L157** | CHEMBL379218 | ZINC000016052569 | 46 |
| L118 | CHEMBL2312191 | ZINC000095593936 | 147 | **L158** | CHEMBL379975 | ZINC000034804008 | 199.53 |
| L119 | CHEMBL2347597 | ZINC000095605236 | 3 | **L159** | CHEMBL3809829 | ZINC000207617458 | 280 |
| L120 | CHEMBL2348641 | ZINC000095603985 | 43 | **L160** | CHEMBL384304 | ZINC000013983251 | 3 |

| Ligand | ChEMBL ID | ZINC ID | Ki |
| --- | --- | --- | --- |
| L161 | CHEMBL385373 | ZINC000014950269 | 451 |
| L162 | CHEMBL388978 | ZINC000003814434 | 4 |
| L163 | CHEMBL393929 | ZINC000028821265 | 340 |
| L164 | CHEMBL396377 | ZINC000006189674 | 2000 |
| L165 | CHEMBL396523 | ZINC000014960327 | 79.43 |
| L166 | CHEMBL412142 | ZINC000029053046 | 1584.89 |
| L167 | CHEMBL413779 | ZINC000014958195 | 1995.26 |
| L168 | CHEMBL41783 | ZINC000013489662 | 1258.93 |
| L169 | CHEMBL428690 | ZINC000021288966 | 190 |
| L170 | CHEMBL431336 | ZINC000013559870 | 30 |
| L171 | CHEMBL434337 | ZINC000013582646 | 6 |
| L172 | CHEMBL442957 | ZINC000013559869 | 140 |
| L173 | CHEMBL443962 | ZINC000040979878 | 251.19 |
| L174 | CHEMBL453217 | ZINC000040845610 | 100 |
| L175 | CHEMBL45408 | ZINC000013559871 | 110 |
| L176 | CHEMBL458997 | ZINC000020148986 | 1995.26 |
| L177 | CHEMBL462120 | ZINC000016158366 | 5011.87 |
| L178 | CHEMBL47302 | ZINC000000141286 | 6500 |
| L179 | CHEMBL475251 | ZINC000006745792 | 125.89 |
| L180 | CHEMBL47527 | ZINC000002047391 | 60 |
| L181 | CHEMBL48109 | ZINC000000603047 | 290 |
| L182 | CHEMBL482967 | ZINC000003950132 | 390 |
| L183 | CHEMBL485731 | ZINC000040938833 | 1500 |
| L184 | CHEMBL491758 | ZINC000000027781 | 398.11 |
| L185 | CHEMBL504950 | ZINC000000069901 | 7943.28 |
| L186 | CHEMBL50894 | ZINC000000023894 | 31.62 |
| L187 | CHEMBL511811 | ZINC000006716290 | 2400 |
| L188 | CHEMBL513846 | ZINC000004007953 | 794.33 |
| L189 | CHEMBL565459 | ZINC000044667946 | 16 |
| L190 | CHEMBL565460 | ZINC000045245785 | 69 |
| L191 | CHEMBL565612 | ZINC000003973984 | 125.89 |
| L192 | CHEMBL570367 | ZINC000045258156 | 1100 |
| L193 | CHEMBL571038 | ZINC000044667947 | 240 |
| L194 | CHEMBL571484 | ZINC000045284232 | 209 |
| L195 | CHEMBL572162 | ZINC000045284641 | 57 |
| L196 | CHEMBL577193 | ZINC000045289749 | 290 |
| L197 | CHEMBL578061 | ZINC000000549484 | 5011.87 |
| L198 | CHEMBL583144 | ZINC000044717144 | 5011.87 |
| L199 | CHEMBL584754 | ZINC000045318107 | 140 |
| L200 | CHEMBL590109 | ZINC000045335993 | 125.89 |
| L201 | CHEMBL592030 | ZINC000045338837 | 12.59 |
| L202 | CHEMBL86066 | ZINC000002573787 | 1584.89 |
