# Supplementary material for: Ensemble learning from ensemble docking: revisiting the optimum ensemble size problem
Source: Sci Rep. 2022 Jan 10;12:410. doi: 10.1038/s41598-021-04448-5 (PMC8748946; doi:10.1038/s41598-021-04448-5)
Supplement: Supplementary file 11 — Supplementary Information 11. [file 41598_2021_4448_MOESM11_ESM.docx]

**Table S4.** Impurity importance of features and total (TOT) importance of chains. The first 84 important chains are listed.

| Chain | TOT | ELC | VHD | INT | TOR | Chain | TOT | ELC | VHD | INT | TOR |
| --- | --- | --- | --- | --- | --- | --- | --- | --- | --- | --- | --- |
| 5ANEA | 18.66 | 17.28 | 0.89 | 0.47 | 0.02 | **3RKBA** | 2.52 | 1.25 | 0.76 | 0.49 | 0.02 |
| 3RZBA | 14.79 | 13.69 | 0.4 | 0.68 | 0.02 | **1FINC** | 2.49 | 0.89 | 0.74 | 0.84 | 0.02 |
| 4ERWA | 9.1 | 6.86 | 1.66 | 0.56 | 0.02 | **3QRUA** | 2.42 | 1.01 | 0.46 | 0.93 | 0.02 |
| 3R9DA | 8.87 | 7.56 | 0.73 | 0.56 | 0.02 | **3QQHA** | 2.38 | 1.64 | 0.27 | 0.45 | 0.02 |
| 3R8UA | 7.6 | 6.28 | 0.75 | 0.54 | 0.03 | **2C5YA** | 2.38 | 0.87 | 0.69 | 0.8 | 0.02 |
| 5JQ8A | 7.03 | 5.24 | 0.88 | 0.89 | 0.02 | **2WEVC** | 2.37 | 1.19 | 0.48 | 0.68 | 0.02 |
| 5A14A | 5.97 | 3.74 | 0.84 | 1.37 | 0.02 | **2WPAC** | 2.32 | 1.02 | 0.43 | 0.85 | 0.02 |
| 3TIZA | 5.11 | 1.63 | 2.42 | 1.04 | 0.02 | **1JVPP** | 2.31 | 1.3 | 0.45 | 0.54 | 0.02 |
| 3TIYA | 5.06 | 4.18 | 0.29 | 0.57 | 0.02 | **3IG7A** | 2.3 | 1.41 | 0.4 | 0.46 | 0.03 |
| 1GY3C | 4.9 | 2.34 | 2.08 | 0.46 | 0.02 | **4EOSA** | 2.29 | 1.33 | 0.53 | 0.41 | 0.02 |
| 3QHWA | 4.64 | 2.94 | 1.23 | 0.45 | 0.02 | **4CFUC** | 2.28 | 0.89 | 0.29 | 1.07 | 0.03 |
| 3QX4A | 4.62 | 3 | 0.66 | 0.94 | 0.02 | **3TNWA** | 2.28 | 1.34 | 0.58 | 0.34 | 0.02 |
| 2R3GA | 4.41 | 3.07 | 0.51 | 0.81 | 0.02 | **2UZBA** | 2.27 | 1.41 | 0.45 | 0.39 | 0.02 |
| 1HCKA | 4.23 | 0.91 | 1.46 | 1.84 | 0.02 | **5IEYA** | 2.25 | 1.42 | 0.38 | 0.43 | 0.02 |
| 2G9XC | 4.16 | 2.88 | 0.76 | 0.5 | 0.02 | **2WIHA** | 2.22 | 0.8 | 0.58 | 0.82 | 0.02 |
| 1FINA | 3.94 | 2.62 | 0.33 | 0.96 | 0.03 | **2C6TC** | 2.21 | 1.36 | 0.37 | 0.46 | 0.02 |
| 1PF8A | 3.86 | 1.36 | 0.71 | 1.77 | 0.02 | **2C5XC** | 2.21 | 0.85 | 0.91 | 0.42 | 0.03 |
| 1FQ1B | 3.63 | 1.42 | 1.78 | 0.41 | 0.02 | **2B54A** | 2.21 | 0.84 | 0.94 | 0.41 | 0.02 |
| 3RJCA | 3.63 | 2.61 | 0.64 | 0.36 | 0.02 | **2W1HA** | 2.19 | 1.28 | 0.37 | 0.52 | 0.02 |
| 3UNJA | 3.58 | 2.53 | 0.41 | 0.62 | 0.02 | **2C4GA** | 2.19 | 1.34 | 0.34 | 0.49 | 0.02 |
| 3RPRA | 3.54 | 2.57 | 0.57 | 0.38 | 0.02 | **3QQGA** | 2.17 | 0.62 | 0.43 | 1.1 | 0.02 |
| 3R9OA | 3.48 | 1.74 | 1.25 | 0.47 | 0.02 | **3F5XC** | 2.14 | 1.14 | 0.52 | 0.46 | 0.02 |
| 3RK5A | 3.36 | 1.79 | 0.95 | 0.6 | 0.02 | **2CCHA** | 2.13 | 1.17 | 0.42 | 0.52 | 0.02 |
| 5IEVA | 3.14 | 1.15 | 1.21 | 0.76 | 0.02 | **5NEVA** | 2.07 | 1 | 0.37 | 0.67 | 0.03 |
| 3R7IA | 3.12 | 1.09 | 1.11 | 0.9 | 0.02 | **4BCOA** | 2.06 | 0.6 | 1 | 0.44 | 0.02 |
| 3PXZA | 2.99 | 1.89 | 0.53 | 0.55 | 0.02 | **3QXPA** | 2.06 | 1.2 | 0.52 | 0.32 | 0.02 |
| 1P5EA | 2.88 | 1.89 | 0.52 | 0.45 | 0.02 | **2UZLC** | 2.04 | 1.07 | 0.33 | 0.62 | 0.02 |
| 3QQLA | 2.82 | 1.79 | 0.63 | 0.37 | 0.03 | **1PXKA** | 2.02 | 1.19 | 0.47 | 0.34 | 0.02 |
| 2C6TA | 2.8 | 1.24 | 0.9 | 0.64 | 0.02 | **3R8MA** | 2 | 1 | 0.63 | 0.35 | 0.02 |
| 3QTRA | 2.78 | 2.01 | 0.33 | 0.42 | 0.02 | **4I3ZA** | 1.99 | 1.14 | 0.35 | 0.48 | 0.02 |
| 3IGGA | 2.78 | 0.6 | 1.15 | 1.01 | 0.02 | **3ULIA** | 1.99 | 0.78 | 0.44 | 0.75 | 0.02 |
| 1DI8A | 2.78 | 1.46 | 0.7 | 0.6 | 0.02 | **3QL8A** | 1.99 | 1.03 | 0.44 | 0.5 | 0.02 |
| 3SW4A | 2.73 | 1.71 | 0.52 | 0.48 | 0.02 | **3QU0A** | 1.97 | 0.86 | 0.54 | 0.55 | 0.02 |
| 3R8VA | 2.69 | 1.86 | 0.34 | 0.47 | 0.02 | **3R8ZA** | 1.96 | 0.97 | 0.62 | 0.35 | 0.02 |
| 3QWKA | 2.64 | 1.47 | 0.39 | 0.76 | 0.02 | **2WIHC** | 1.96 | 0.52 | 0.38 | 1.04 | 0.02 |
| 2C5NA | 2.6 | 0.74 | 1.43 | 0.41 | 0.02 | **3PXQA** | 1.94 | 1.13 | 0.31 | 0.48 | 0.02 |
| 1PXNA | 2.58 | 1.4 | 0.27 | 0.88 | 0.03 | **4BCNC** | 1.94 | 0.65 | 0.67 | 0.6 | 0.02 |
| 4CFWA | 2.57 | 0.94 | 0.96 | 0.65 | 0.02 | **2VTRA** | 1.94 | 1.06 | 0.38 | 0.48 | 0.02 |
| 1VYWC | 2.56 | 1.4 | 0.43 | 0.71 | 0.02 | **1KE7A** | 1.94 | 0.82 | 0.52 | 0.58 | 0.02 |
| 2CCIA | 2.56 | 0.77 | 1.2 | 0.57 | 0.02 | **2XNBA** | 1.92 | 0.86 | 0.5 | 0.54 | 0.02 |
| 3BHUA | 2.54 | 1.43 | 0.23 | 0.86 | 0.02 | **3QZHA** | 1.9 | 0.82 | 0.49 | 0.57 | 0.02 |
| 2FVDA | 2.54 | 1.44 | 0.68 | 0.4 | 0.02 | **3QRTA** | 1.9 | 0.61 | 0.47 | 0.8 | 0.02 |
