# Supplementary material for: Ensemble learning from ensemble docking: revisiting the optimum ensemble size problem
Source: Sci Rep. 2022 Jan 10;12:410. doi: 10.1038/s41598-021-04448-5 (PMC8748946; doi:10.1038/s41598-021-04448-5)
Supplement: Supplementary file 12 — Supplementary Information 12. [file 41598_2021_4448_MOESM12_ESM.docx]

**Table S5.** Permutation importance of features and total (TOT) importance of chains. The first 84 important chains are listed.

| Chain | TOT | ELC | VHD | INT | TOR | Chain | TOT | ELC | VHD | INT | TOR |
| --- | --- | --- | --- | --- | --- | --- | --- | --- | --- | --- | --- |
| 5ANEA | 11.24 | 8.91 | 1.64 | 0.72 | -0.03 | **3SW4A** | 3.51 | 1.78 | 1.19 | 0.47 | 0.07 |
| 3RZBA | 9.45 | 7.34 | 0.98 | 1.25 | -0.12 | **3QTRA** | 3.48 | 1.72 | 0.92 | 0.84 | 0.00 |
| 4ERWA | 7.36 | 4.89 | 2.18 | 0.23 | 0.06 | **1OIYA** | 3.47 | 0.97 | 1.4 | 0.78 | 0.32 |
| 3QX4A | 7.27 | 3.69 | 1.88 | 1.51 | 0.19 | **1VYWC** | 3.46 | 1.14 | 0.94 | 1.07 | 0.31 |
| 5JQ8A | 6.98 | 3.6 | 1.74 | 1.33 | 0.31 | **2WPAC** | 3.41 | 0.63 | 0.55 | 1.84 | 0.39 |
| 3R8UA | 6.95 | 4.04 | 1.64 | 1.01 | 0.26 | **2CCIA** | 3.37 | 0.39 | 1.94 | 1.08 | -0.04 |
| 5A14A | 6.70 | 3.06 | 1.31 | 2.21 | 0.12 | **3TNWA** | 3.35 | 1.58 | 1.31 | 0.35 | 0.11 |
| 3R9DA | 6.69 | 5.10 | 1.27 | 0.04 | 0.28 | **3RKBA** | 3.34 | 1.35 | 1.61 | 0.32 | 0.06 |
| 1GY3C | 6.54 | 3.20 | 2.67 | 0.71 | -0.04 | **2UZLC** | 3.33 | 0.9 | 0.87 | 1.25 | 0.31 |
| 3TIZA | 6.09 | 1.40 | 3.12 | 1.42 | 0.15 | **2C5VC** | 3.32 | 1.44 | 0.37 | 1.27 | 0.24 |
| 1FINA | 5.98 | 3.49 | 0.67 | 1.81 | 0.01 | **2C6TC** | 3.28 | 1.3 | 0.9 | 1.01 | 0.07 |
| 3TIYA | 5.69 | 3.23 | 0.91 | 1.15 | 0.4 | **2XNBA** | 3.26 | 0.76 | 1.62 | 0.75 | 0.13 |
| 3R9OA | 5.67 | 2.47 | 2.27 | 0.47 | 0.46 | **2UZDA** | 3.26 | 0.63 | 1.94 | 0.63 | 0.06 |
| 1PF8A | 5.49 | 1.73 | 1.43 | 2.39 | -0.06 | **3IG7A** | 3.23 | 1.25 | 1.05 | 0.95 | -0.02 |
| 2R3GA | 5.29 | 2.85 | 0.88 | 1.22 | 0.34 | **4CFUC** | 3.2 | 0.56 | 0.82 | 1.73 | 0.09 |
| 2C6TA | 5.29 | 1.6 | 2.08 | 1.57 | 0.04 | **2VTRA** | 3.2 | 1.52 | 0.98 | 0.67 | 0.03 |
| 2G9XC | 5.18 | 2.45 | 1.85 | 0.78 | 0.1 | **4BCOA** | 3.19 | 0.39 | 1.71 | 0.92 | 0.17 |
| 5IEVA | 5.17 | 1.17 | 2.53 | 1.23 | 0.24 | **2UZEC** | 3.18 | 0.93 | 0.94 | 1.11 | 0.20 |
| 3R7IA | 5.17 | 0.76 | 2.35 | 1.59 | 0.47 | **2C5NA** | 3.17 | -0.1 | 2.49 | 0.56 | 0.22 |
| 3QHWA | 5.04 | 2.18 | 2.06 | 0.67 | 0.13 | **2WEVC** | 3.16 | 0.86 | 1.09 | 1.24 | -0.03 |
| 1HCKA | 4.73 | 0.43 | 2.07 | 2.32 | -0.09 | **1P5EC** | 3.16 | 0.62 | 0.62 | 1.62 | 0.30 |
| 3QQLA | 4.71 | 2.05 | 1.29 | 0.95 | 0.42 | **3QZHA** | 3.09 | 0.83 | 1.07 | 1.12 | 0.07 |
| 1DI8A | 4.67 | 1.78 | 1.69 | 1.02 | 0.18 | **2WIHC** | 3.05 | 0.39 | 0.92 | 1.67 | 0.07 |
| 4CFWA | 4.64 | 1.24 | 1.86 | 1.35 | 0.19 | **2UZBA** | 3.04 | 0.94 | 1.12 | 0.81 | 0.17 |
| 3RK5A | 4.53 | 1.97 | 1.92 | 0.77 | -0.13 | **3QWKA** | 3.02 | 1.11 | 0.7 | 1.15 | 0.06 |
| 1FQ1B | 4.53 | 1.16 | 2.66 | 0.55 | 0.16 | **4II5C** | 3.02 | 0.86 | 0.99 | 1.04 | 0.13 |
| 2CCHA | 4.25 | 1.59 | 1.13 | 1.03 | 0.5 | **2UUEA** | 3 | 0.41 | 0.77 | 1.47 | 0.35 |
| 1P5EA | 4.2 | 2.16 | 0.87 | 0.79 | 0.38 | **5NEVA** | 2.98 | 0.58 | 0.79 | 1.69 | -0.08 |
| 3UNJA | 4.18 | 1.98 | 1.39 | 0.46 | 0.35 | **1PXNA** | 2.96 | 1.32 | 0.42 | 0.92 | 0.3 |
| 2FVDA | 4.14 | 1.34 | 1.62 | 0.89 | 0.29 | **2C5XC** | 2.94 | 0.36 | 1.93 | 0.55 | 0.10 |
| 3IGGA | 4.12 | 0.34 | 2.01 | 1.74 | 0.03 | **4EZ3A** | 2.94 | 0.38 | 1.47 | 0.83 | 0.26 |
| 3QRUA | 4.03 | 1.3 | 0.89 | 1.68 | 0.16 | **1FINC** | 2.93 | 0.78 | 1.28 | 0.85 | 0.02 |
| 2WIHA | 3.92 | 0.75 | 1.28 | 1.91 | -0.02 | **4EOSC** | 2.92 | 0.25 | 1.18 | 1.4 | 0.09 |
| 2C4GA | 3.92 | 1.93 | 0.49 | 1.11 | 0.39 | **3RNIA** | 2.92 | 0.71 | 1.44 | 0.53 | 0.24 |
| 2C5YA | 3.91 | 0.73 | 1.84 | 1.18 | 0.16 | **3RPRA** | 2.89 | 2.07 | 0.82 | 0.08 | -0.08 |
| 3R8VA | 3.87 | 2.29 | 0.82 | 0.45 | 0.31 | **1JVPP** | 2.84 | 1.27 | 0.72 | 0.82 | 0.03 |
| 3BHUA | 3.86 | 1.78 | 0.45 | 1.29 | 0.34 | **1JSTC** | 2.82 | 0.41 | 1.28 | 0.87 | 0.26 |
| 3RJCA | 3.66 | 2.26 | 1.44 | 0.05 | -0.09 | **3F5XC** | 2.77 | 0.68 | 0.94 | 0.91 | 0.24 |
| 3PXZA | 3.66 | 2.1 | 1.06 | 0.36 | 0.14 | **5IEYA** | 2.75 | 0.91 | 0.87 | 0.77 | 0.2 |
| 3ULIA | 3.59 | 0.74 | 1.33 | 1.4 | 0.12 | **4EORA** | 2.7 | 1.02 | 0.88 | 0.56 | 0.24 |
| 3QXPA | 3.55 | 1.39 | 1.26 | 0.7 | 0.2 | **1OIUA** | 2.69 | 0.74 | 1.32 | 0.53 | 0.10 |
| 2B54A | 3.53 | 0.65 | 1.84 | 0.86 | 0.18 | **3R8ZA** | 2.68 | 0.4 | 1.36 | 0.74 | 0.18 |
